# Importance of the N-Terminal Domain of the Qb-SNARE Vti1p for Different Membrane Transport Steps in the Yeast Endosomal System

**DOI:** 10.1371/journal.pone.0066304

**Published:** 2013-06-12

**Authors:** Michael Gossing, Subbulakshmi Chidambaram, Gabriele Fischer von Mollard

**Affiliations:** Biochemie III, Fakultät für Chemie, Universität Bielefeld, Bielefeld, Germany; Institut Curie, France

## Abstract

SNAREs (soluble N-ethylmaleimide-sensitive factor attachment protein receptor) on transport vesicles and target membranes are crucial for vesicle targeting and fusion. They form SNARE complexes, which contain four α-helical SNARE motifs contributed by three or four different SNAREs. Most SNAREs function only in a single transport step. The yeast SNARE Vti1p participates in four distinct SNARE complexes in transport from the trans Golgi network to late endosomes, in transport to the vacuole, in retrograde transport from endosomes to the trans Golgi network and in retrograde transport within the Golgi. So far, all *vti1* mutants investigated had mutations within the SNARE motif. Little is known about the function of the N-terminal domain of Vti1p, which forms a three helix bundle called H_abc_ domain. Here we generated a temperature-sensitive mutant of this domain to study the effects on different transport steps. The secondary structure of wild type and *vti1-3* H_abc_ domain was analyzed by circular dichroism spectroscopy. The amino acid exchanges identified in the temperature-sensitive *vti1-3* mutant caused unfolding of the H_abc_ domain. Transport pathways were investigated by immunoprecipitation of newly synthesized proteins after pulse-chase labeling and by fluorescence microscopy of a GFP-tagged protein cycling between plasma membrane, early endosomes and Golgi. In *vti1-3* cells transport to the late endosome and assembly of the late endosomal SNARE complex was blocked at 37°C. Retrograde transport to the trans Golgi network was affected while fusion with the vacuole was possible but slower. Steady state levels of SNARE complexes mediating these steps were less affected than that of the late endosomal SNARE complex. As different transport steps were affected our data demonstrate the importance of a folded Vti1p H_abc_ domain for transport.

## Introduction

SNARE proteins are key players in membrane fusion in eukaryotic organisms. Localized on opposing membranes, four SNARE motifs of three to four SNAREs interact to form a SNARE complex, thus bringing the membranes in close proximity and facilitating fusion [1]. The SNARE complex consists of an α-helical four-helix-bundle, which forms 16 layers of interacting amino acids. These layers are mainly hydrophobic, with the exception of the central 0-layer, which is comprised of three glutamines (Q) and one arginine (R). Depending on the amino acid in the 0-layer, SNAREs are divided into Q- and R-SNAREs. A further subdivision of Q-SNAREs into Q_a_-, Q_b_- and Q_c_-SNAREs is based on similarities in the amino acid sequence of their SNARE motifs. SNARE complexes are universally formed by four different SNARE motifs, one of each type, yielding a Q_a_Q_b_Q_c_R-SNARE complex. 25 SNAREs have been identified in yeast and more than 40 in mammalian cells. SNARE complexes have an organelle-specific composition to mediate transport between different subcellular compartments [2]. Some SNARE proteins participate in the formation of multiple different SNARE complexes. The yeast Q_b_-SNARE Vti1p is part of four different SNARE complexes. At the late endosome (prevacuolar compartment), fusion is mediated by a SNARE complex consisting of Pep12p (Q_a_), Vti1p (Q_b_), Syn8p (Q_c_) and Ykt6p (R) [3], [4], [5]. Vacuolar fusion events are mediated by a SNARE complex formed by Vam3p (Q_a_), Vti1p (Q_b_), Vam7p (Q_c_) and Nyv1p (R) or Ykt6p (R) [6]. Homotypic fusion of the trans Golgi network (TGN) and retrograde transport from early endosomes to the TGN are mediated by a SNARE complex formed by Tlg2p (Q_a_), Vti1p (Q_b_), Tlg1p (Q_c_) and Snc1p or Snc2p (R) [7], [8]. Intra-Golgi fusion events are carried out by a SNARE complex composed of Sed5p (Q_a_), Vti1p or Gos1p (Q_b_), Sft1p (Q_c_) and Ykt6p (R) [9], [10], [11].

In addition to their SNARE motif, SNAREs often feature autonomously folded N-terminal domains, which exhibit a variety of functions, including recruitment of tethering proteins and regulatory factors as well as correct sorting of the SNARE protein. It is particularly their regulatory role which has created much interest. The N-terminal domains of all Q_a_-SNAREs and many Q_b_- and Q_c_- SNAREs consist of a H_abc_ domain, which is formed by an antiparallel three-helix-bundle. The N-terminal domains of R-SNAREs fall into two subcategories, the first being the brevins, which possess a short N-terminus, and the second being the longins, which possess a longin domain with a profilin-like fold. Some Q_a_- and Longin R-SNAREs can adopt a closed conformation. This conformation is formed by folding of the N-terminal domain onto the SNARE motif, yielding a fusion-incompetent SNARE. Sec1/Munc18 (SM) proteins form a small family of soluble proteins that are important regulators of membrane fusion with four family members in yeast and seven family members in higher eukarya [12]. Some SM proteins interact with the SNARE machinery by binding to N-terminal domains of Q_a_-SNAREs. The SM proteins Sly1p and Vps45p bind to the Q_a_-SNAREs Ufe1p, Sed5p and Tlg2p via a N-terminal peptide motif [13], [14]. Less is known about the role of the N-terminal domains of Q_b_- and Q_c_-SNAREs in vesicle trafficking. The N-termini of the Q_b_-SNARE Sec20p and the Q_c_-SNARE Use1p interact with Tip20p and Dsl3p, respectively, which are components of the Dsl1 tethering complex [15], [16]. Tethering complexes are responsible for the initial contact between transport vesicle and target membrane. The H_abc_ domain of the Q_c_-SNARE Tlg1p interacts with Vps51p, a component of the GARP tethering complex [17].

The N-terminal domains of Vti1p and its mammalian homologs, vti1a and vti1b, form a H_abc_ domain. No evidence was obtained for a closed-conformation of vti1b [18]. In earlier studies, we demonstrated that Vti1p and vti1b interact with the Epsin N-terminal homology (ENTH) domain proteins Ent3p and EpsinR, respectively [19], which mediates sorting into clathrin coated vesicles and correct localization *in vivo* [20], [21]. However, despite mislocalization of late endosomal SNAREs in *ent3*Δ cells, transport from the Golgi to the late endosome is almost normal. This transport step is only slightly affected in the absence of Ent3p and Ent5p, which has partially overlapping functions with Ent3p but does not bind Vti1p [19]. In this study, we investigated additional functions of the Vti1p H_abc_ domain in vesicle trafficking.

## Results

### Effects of Mutations in the Vti1p N-terminus on Different Vesicle Traffic Steps

The aim of this work is to analyze whether the N-terminal domain of Vti1p has functions in different vesicle traffic steps. We selected transport of carboxypeptidase Y (CPY) and alkaline phosphatase (ALP) and recycling of GFP-Snc1p to analyze three different transport pathways.

**Figure 1:**
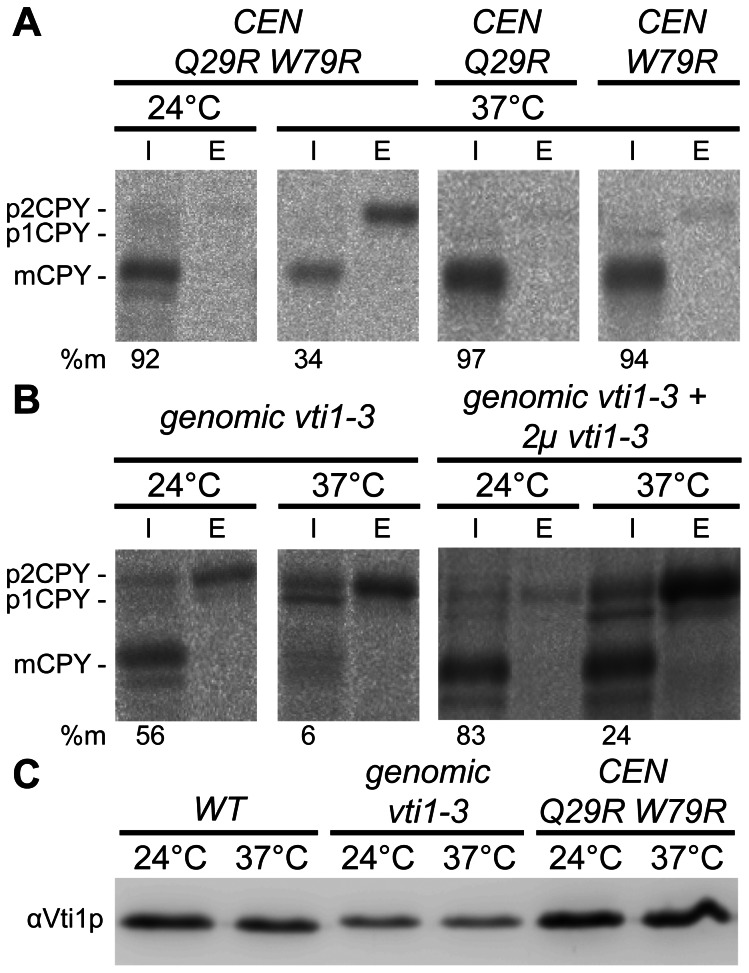
Defective CPY sorting in *vti1-3* cells. CPY (carboxypeptidase Y) sorting was analyzed by pulse-chase labeling and CPY immunoprecipitations from cellular extracts (I, intracellular) and medium (E, extracellular). The numbers are percentage of vacuolar mCPY in the experiment shown. (A) The N-terminal double mutant Vti1p Q29R W79R secreted Golgi-modified p2CPY at 37°C but not at 24°C. The corresponding single mutants Q29R and W79R displayed no sorting phenotype at 37°C. Strains used: FvMY6 pBK120 (*CEN* Q29R W79R), FvMY6 pBK123 (*CEN* Q29R), FvMY6 pBK128 (*CEN* W79R). (B) Genomic integration of the *vti1-3* mutant resulted in an aggravated CPY missorting. Some Golgi-modified p2CPY was secreted at 24°C. At 37°C, ER-modified p1CPY and Golgi-modified p2CPY accumulated intracellularly, Golgi-modified p2CPY was secreted and very little vacuolar mCPY was detected. Overproduction of *vti1-3p* reduced the CPY sorting defect at 24°C. Strains used: SCY14 (*vti1-3*), SCY14 pSC14 (*vti1-3*+2µ *vti1-3*). (C) Vti1p or *vti1-3p* protein levels in different strains at 24°C or after 2 h at 37°C detected by western blotting using antiserum against Vti1p.

To be able to observe acute defects we screened for temperature-sensitive N-terminal mutants of Vti1p. Random mutagenesis of *VTI1* on a *CEN*-based plasmid generated a mutant (*vti1-3*) with two mutations (Q29R W79R) in the H_abc_ domain of Vti1p. The effect of these amino acid exchanges on CPY sorting was studied by immunoprecipitations after pulse-chase labeling. The soluble vacuolar protease CPY is transported to the vacuole via the secretory pathway and the prevacuolar compartment (late endosome) [22]. CPY exists in three different forms, an ER-modified form of 67 kDa (p1CPY), a Golgi-modified form of 69 kDa (p2CPY) and a vacuolar, mature form of 61 kDa (mCPY). A block in trafficking between the TGN and the late endosome causes secretion of p2CPY. At 24°C, the majority of CPY was correctly transported to the vacuole (mCPY: 92±1.3%, n = 2, Fig. 1A). At 37°C, Golgi-modified p2CPY was secreted (mCPY: 32±13.3%, n = 7), indicating a temperature-dependent sorting defect. These data suggest a role of the Vti1p H_abc_ domain in transport of CPY. Variants with the single amino acid exchanges Q29R or W79R, respectively, were constructed. They displayed no sorting defect at 37°C (mCPY: 89±11%, n = 2 and 93±1.6%, n = 2, respectively, Fig. 1A). The *vti1-3* mutation was integrated into the genome for further analyses. The CPY sorting defect of the genomically integrated mutant was aggravated (Fig. 1B). Some Golgi-modified p2CPY was already secreted at 24°C (mCPY: 51±9.4%, n = 3). At 37°C, ER-modified p1CPY and Golgi-modified p2CPY accumulated within the cell, Golgi-modified p2CPY was secreted and very little CPY reached the vacuole (mCPY: 6±1.2%, n = 3). The accumulation of p1CPY suggests that glycosylation in the Golgi apparatus is not fully functional due to missorting of some Golgi localized glycosylation enzymes as observed in *vti1-11* and *vti1-22* cells [10], [23]. The worsening of the CPY sorting defect was due to reduced *vti1-3p* protein levels upon genomic integration (Fig. 1C). Protein levels of *vti1-3p* expressed from a *CEN*-based plasmid were comparable to wild type levels of Vti1p (*CEN vti1-3,* 24°C: 122±19, 37°C: 118±14, n = 3). By contrast, upon genomic integration, levels of *vti1-3p* are reduced to 49±10% at 24°C and 43±12% at 37°C (n = 6). A multicopy *vti1-3* plasmid was introduced into yeast cells expressing genomic *vti1-3* to increase the levels of *vti1-3p* to more than twice the levels of wild type Vti1p (data not shown). This overexpression of *vti1-3p* resulted in a CPY sorting defect comparable to that in cells expressing *vti1-3* from a *CEN*-based plasmid (Fig. 1B). These data demonstrate that the amino acid exchanges Q29R W79R in the H_abc_ domain caused a CPY transport defect that could not be corrected by overexpression of this mutant protein.

**Figure 2:**
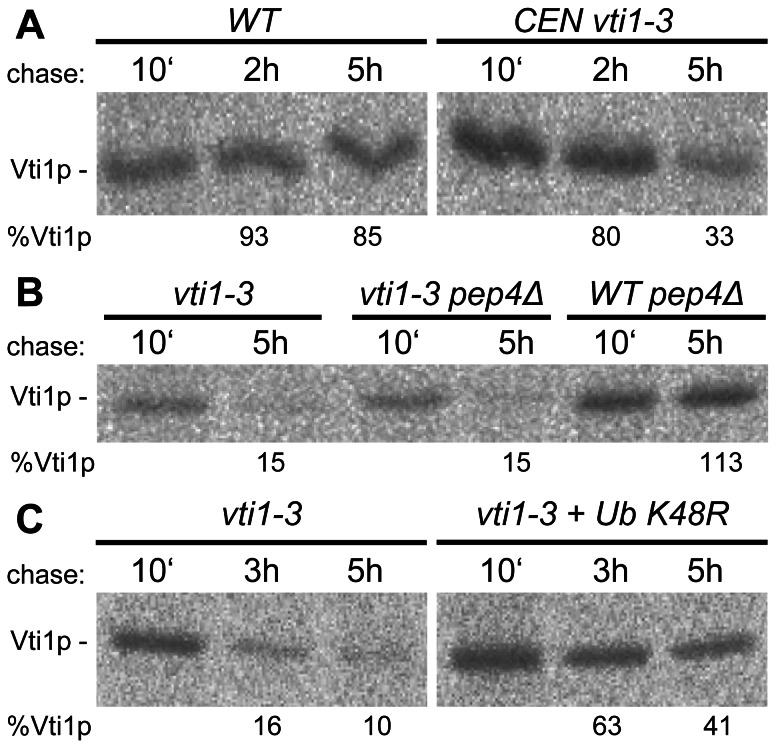
Analyses of stability of *vti1-3p*. Stability of *vti1-3p* was analyzed by pulse-chase labeling at 37°C and immunoprecipitation of Vti1p. (A) Stability of *vti1-3p* was reduced compared to wild type. Cells were labeled with [^35^S]methionine and [^35^S]cysteine for 25 min and chased for the indicated duration. Vti1p was immunoprecipitated from the cellular extracts and the percentage of Vti1p remaining after 2 and 5 hours was calculated relative to that at 10 min. Strains used: SEY6211 (WT), FvMY6 pBK120 (*CEN vti1-3*). (B) *vti1-3p* was not stabilized in *pep4Δ* cells deficient for vacuolar proteases in contrast to Vti1p. Strains used: SCY14 (*vti1-3*), SCY18 (*vti1-3 pep4Δ*), BKY12 (WT *pep4Δ*). (C) *vti1-3p* was stabilized in cells expressing ubiquitin K48R. Strains used: SCY14 (*vti1-3*), SCY14 pUB203 (*vti1-3+ Ub K48R*).

The amino acid exchanges in the H_abc_ domain might affect protein stability. Therefore, we investigated stability of *vti1-3p* by immunoprecipitation after pulse-chase labeling at 37°C (Fig. 2). *vti1-3p* expressed from a *CEN*-based plasmid was degraded faster than wild type Vti1p (Fig. 2A). Stability of genomically integrated *vti1-3p* was even further reduced (Fig. 2B). To gather insight into the pathway of *vti1-3p* degradation, we analyzed stability of *vti1-3p* in cells that do not display vacuolar proteolytic activity (*pep4Δ*). (Fig. 2B), No stabilization of *vti1-3p* in *pep4Δ* cells was observed in contrast to wild type Vti1p. To investigate the role of proteasomal degradation, the stability of *vti1-3p* was analyzed in *vti1-3* cells overexpressing ubiquitin K48R, a mutant that prevents polyubiquitination and thus proteasomal degradation (Fig. 2C). In the presence of Ubiquitin K48R, *vti1-3p* was strongly stabilized. Taken together, these data indicate that *vti1-3p* is degraded by the proteasome, but not in the vacuole. Localization of *vti1-3p* was analyzed by indirect immunofluorescence to assess whether transport defects are due to mislocalization of *vti1-3p*. Similar to wild type Vti1p, *vti1-3p* showed a punctuate distribution in the cell. In agreement with lower protein levels, the intensity of the staining appeared somewhat weaker. The *vti1-3p* staining was slightly more diffuse at 24°C and after 30 min at 37°C compared to wild type cells. However, similar to wild type cells, some colocalization of *vti1-3p* with the endosomal marker protein Vps45p-3HA was observed at both temperatures (Fig. S1).

**Figure 3:**
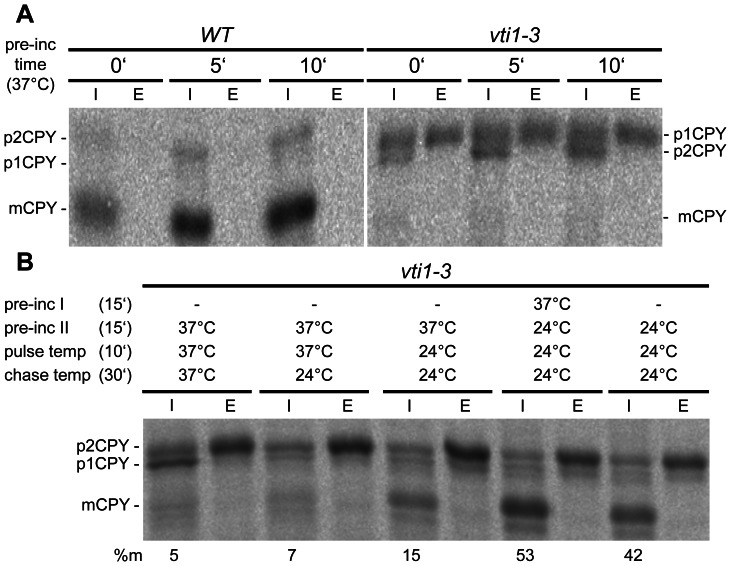
Kinetics of onset and recovery of the CPY sorting defect. CPY sorting was analyzed by pulse-chase labeling and CPY immunoprecipitations from cellular extracts (I, intracellular) and medium (E, extracellular). (A) *vti1-3* cells showed a fast onset of the CPY sorting defect. Reducing preincubation time from 15 min to 10 min, 5 min or 0 min at 37°C did not influence CPY missorting. (B) Transport to the vacuole recovered only slowly after return to 24°C. A full recovery from the vacuolar sorting defect was achieved when cells were preincubated for 15 min at 24°C after previous preincubation at 37°C. Accumulation of ER-modified p1CPY observed at 37°C was already reversed during a 24°C chase after labeling at 37°C. Strain used: SCY14 (*vti1-3*).

To gather mechanistic insights we analyzed the kinetics of onset and reversibility of the observed sorting defect in *vti1-3* cells. The onset kinetics of the sorting defect was investigated by reducing or eliminating the preincubation time at 37°C. *vti1-3* cells showed a rapid onset of the CPY sorting defect (Fig. 3A). Even elimination of the preincubation time at 37°C resulted in a complete block of CPY transport to the vacuole via the late endosome. This rapid onset together with the immunofluorescence results suggest that mislocalization of *vti1-3p* is not the main reason for this defect. Next the kinetics of the recovery from the sorting defect was analyzed. Transport to the vacuole recovered only slowly after return to 24°C (Fig. 3B). A shift to 24°C after labeling at 37°C did not result in a significant increase in mCPY (mCPY: 7±1.8%, n = 3) compared to a chase at 37°C. Shifting the cells to 24°C before labeling led to a small increase in mCPY (mCPY: 19±2.4%, n = 3), while a full recovery from the vacuolar sorting defect was achieved when cells were preincubated for 15 min at 24°C after a previous preincubation at 37°C (mCPY: 59±5.9%, n = 3). By contrast, the accumulation of ER-modified p1CPY observed at 37°C was already reversed during a 24°C chase after labeling at 37°C. These data indicate that the recovery of Golgi sorting was quicker than that of vacuolar transport in *vti1-3* cells.

**Figure 4:**
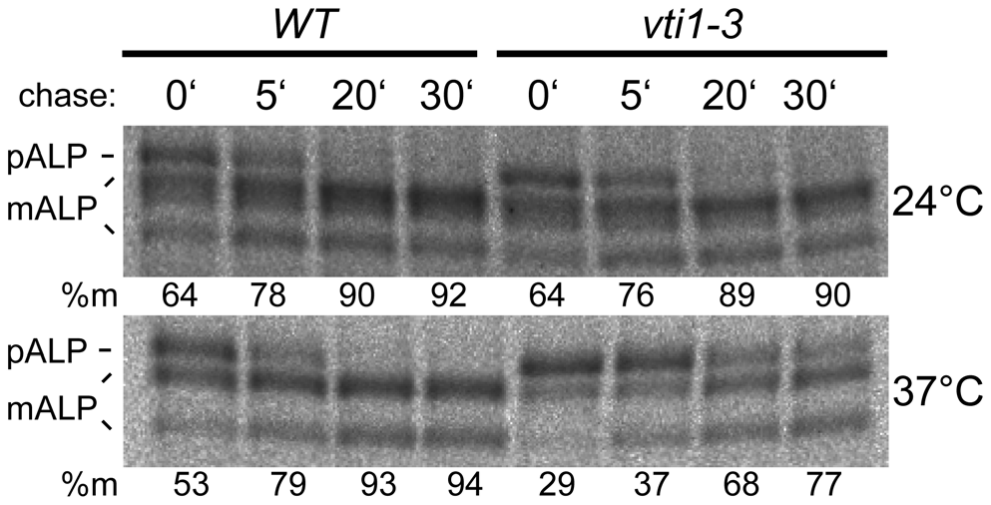
ALP transport was slower in *vti1-3* cells. ALP (alkaline phosphatase) sorting was analyzed by pulse-chase labeling and ALP immunoprecipitations from cellular extracts. The ER and Golgi pro form pALP is processed to vacuolar mature mALP. ALP reached the vacuole with wild type kinetics in *vti1-3* cells at 24°C but not at 37°C. Strains used: SEY6210 (WT), SCY14 (*vti1-3*).

The effect of the mutations on transport directly to the vacuole [22] was analyzed by immunoprecipitations of ALP after pulse-chase labeling (Fig. 4). The vacuolar alkaline phosphatase ALP is a type II integral membrane protein, which traffics via the secretory pathway as ER- and Golgi-modified forms of 76 kDa (pALP). It is transported from the TGN without passing through late endosomes directly to the vacuole where it is processed to a vacuolar, mature form of 72 kDa (mALP) and a smaller product. A block in this transport step between the TGN and the vacuole is indicated by detectable pALP. ALP was transported more slowly to the vacuole in *vti1-3* cells compared to wild type cells at 37°C (Fig. 4) as indicated by lower amounts of mALP directly after the pulse (31±3.6%, n = 2 in *vti1-3* and 60±9.8% in WT cells) and after a chase period of 5 min (41±5.1% in *vti1-3* and 81±3.2% in WT cells). After longer chase periods increasing amounts of ALP reached the vacuole in *vti1-3* cells resulting in 79±3.5% (n = 3) of mALP after 30 min chase. These data indicate that a defective H_abc_ domain resulted predominantly in a kinetic delay in transport to the vacuole.

**Figure 5:**
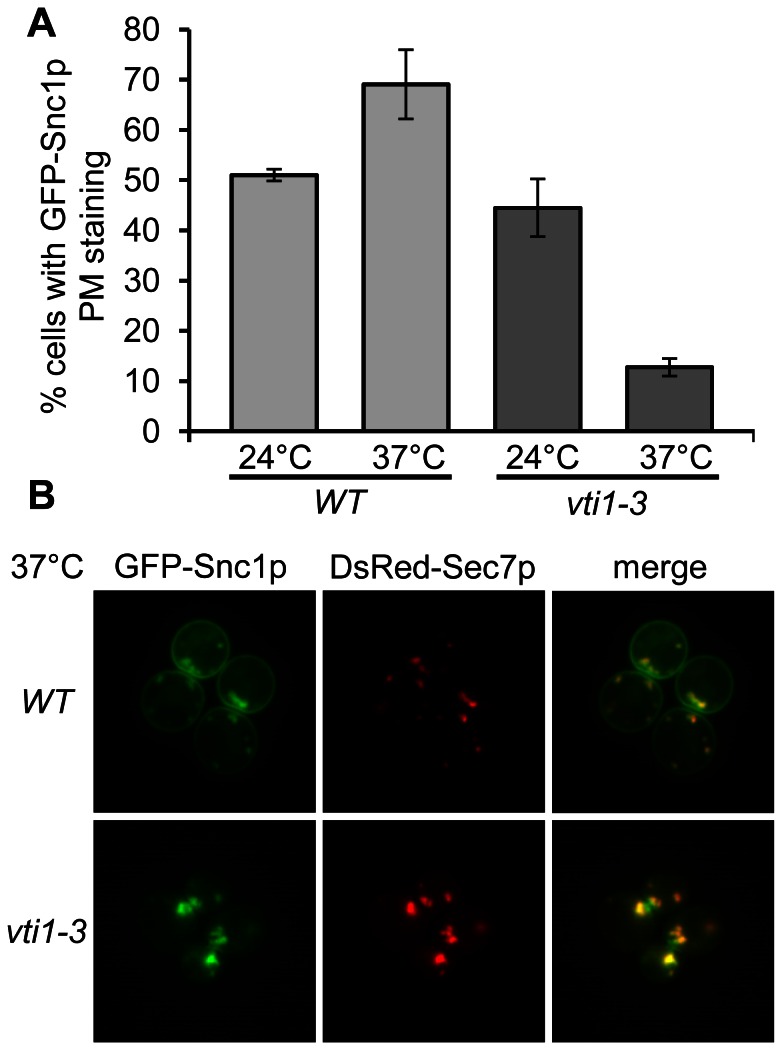
Defective GFP-Snc1p recycling in *vti1-3* cells. (A) GFP-Snc1p recycling was defective in *vti1-3* cells at 37°C. At least 200 cells each were counted in three independent experiments and results are means ± S.D. Strains used: SEY6210 pJJ8 (WT), SCY14 pJJ8 (*vti1-3*). (B) GFP-Snc1p accumulated in intracellular spots, which colocalize with DsRed-Sec7p. Strains used: SEY6210 pJJ8 pTPQ128 (WT), SCY14 pJJ8 pTPQ128 (*vti1-3*). PM plasma membrane.

To analyze transport from endosomes to the TGN, the effect of the mutations on GFP-Snc1p recycling was studied by a fluorescence microscopy approach. Snc1p is an exocytic R-SNARE which mediates fusion of TGN-derived secretory vesicles with the plasma membrane by forming a complex with the Q_a_-SNARE Sso1p or Sso2p and the Q_bc_-SNARE Sec9p [24]. At steady state, Snc1p is predominantly localized to the plasma membrane and it is recycled via early endosomes to the TGN [25]. A block in retrograde transport to the TGN is indicated by a loss of localization to the plasma membrane and intracellular accumulation of Snc1p, which can be assayed by fluorescence microscopy of cells in early log-phase expressing GFP-Snc1p. In wild type cells, GFP-Snc1p was localized to the plasma membrane in 51% (±1.2% in three independent experiments with at least 200 cells each) of the cells at 24°C and 69% ±6.9% at 37°C (Fig. 5A). Higher growth rates at 37°C requiring more exocytic membrane traffic may cause the increased presence of GFP-Snc1p at the plasma membrane. In *vti1-3* cells, GFP-Snc1p was localized to the plasma membrane in 45% (±5.7) of the cells at 24°C, which was reduced to 13% (±1.7%) at 37°C. Upon block of recycling to the TGN, GFP-Snc1p is thought to accumulate in early endosomes and transport vesicles mediating traffic between early endosomes and TGN. These structures also contain TGN proteins because TGN proteins cycle between the TGN and early endosomes. DsRed-Sec7p was expressed as a TGN marker protein [26]. In wild type and *vti1-3* cells, the majority of GFP-Snc1p was localized to the plasma membrane at 24°C, while some GFP-Snc1p was detectable in intracellular structures which colocalized with DsRed-Sec7p (Fig. S2). At 37°C, GFP-Snc1p accumulated in intracellular structures in *vti1-3* cells which colocalized with DsRed-Sec7p (Fig. 5B, S2). This was accompanied by a loss of plasma membrane staining. The colocalization between DsRed-Sec7p and GFP-Snc1p could be due to mislocalization of DsRed-Sec7p to an early endosomal compartment or due to tethering of GFP-Snc1p-containing transport vesicles to the TGN.

### Analyses of SNARE Complex Assembly

**Figure 6:**
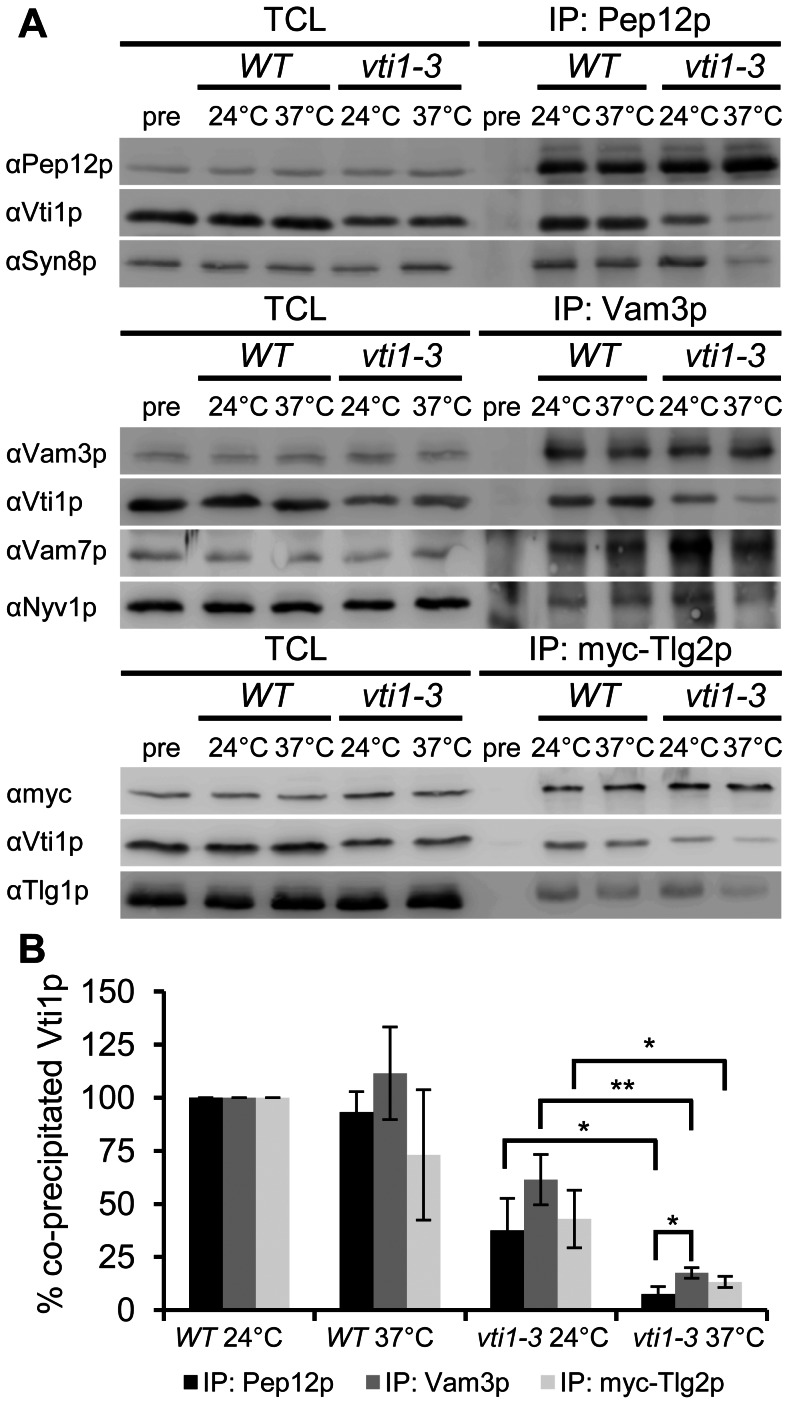
Analyses of SNARE complex assembly by coimmunoprecipitation. (A) *vti1-3p* and Syn8p did not coprecipitate with Pep12p in *vti1-3* cells at 37°C. Strains used: SSY4 (WT), SCY18 (*vti1-3*). Reduced levels of *vti1-3p* coprecipitation with Vam3p were observed in *vti1-3* cells at 37°C. Strains used: SSY4 (WT), SCY18 (*vti1-3*). Vti1p coprecipitated with myc-Tlg2p in *vti1-3* cells at 37°C. Reduced levels of *vti1-3p* coprecipitation were observed in *vti1-3* cells at 37°C. Strains used: SSY4 pMG50 (WT), SCY18 pMG50 (*vti1-3*). TCL: 6.3% of total cell lysate for all panels; IP: 100% of immunoprecipitate. (B) *vti1-3p* was found in SNARE complexes in significantly lower amounts at 37°C compared to 24°C. Western blots were quantified, coprecipitated Vti1p normalized to the amount of Pep12p, Vam3p or myc-Tlg2p, respectively, and Vti1p isolated at 24°C set to 100%. Three independent experiments were quantified. Mean ± standard deviation, **P<0.01, *P<0.05, unpaired Student’s t test.

The observed defects in different vesicle traffic steps prompted us to analyze SNARE complex assembly in wild type and *vti1-3* cells. Assembly of SNARE complexes was studied by coimmunoprecipitations. Pep12p is a Q_a_-SNARE that is essential for all known fusion reactions with the late endosome. It forms a SNARE complex with the Q_b_-SNARE Vti1p, the Q_c_-SNARE Syn8p and the R-SNARE Ykt6p [3], [4], [5]. In wild type cells grown at 24°C, Vti1p coprecipitated with Pep12p after a preincubation for 1 h at 24°C or at 37°C (Fig. 6A). It should be noted that only a small fraction of Vti1p is found in a complex with Pep12p as the amount of total cell lysates (TCL) loaded for all panels of Fig. 6A was only 6.3% of the load of the immunoprecipitates (IP). In *vti1-3* cells, Vti1p coprecipitated with Pep12p at 24°C (37% ±15% compared to Vti1p coprecipitated at 24°C, n = 3), but not at 37°C (7.6% ±3.4%, Fig. 6B). In addition, Syn8p was not coimmunoprecipitated either at 37°C but present at 24°C. These data indicate that the SNARE complex was able to assemble correctly at 24°C, but not at 37°C. The failure of SNARE complex assembly is in agreement with the observed temperature-dependent CPY sorting defect.

Vam3p is a Q_a_-SNARE that mediates fusion at the vacuole. It forms a SNARE complex with the Qb-SNARE Vti1p, the Qc-SNARE Vam7p and the R-SNAREs Ykt6p or Nyv1p [6]. In wild type and *vti1-3* cells, Vti1p coprecipitated with Vam3p at 24°C (Fig. 6A). Reduced levels of coprecipitation were observed in *vti1-3* cells at 37°C (18% ±2.5% compared to Vti1p coprecipitated at 24°C, n = 3). However, significantly more *vti1-3p* coprecipitated with Vam3p than with Pep12p. Coprecipitation of Vam7p and Nyv1p were somewhat reduced at 37°C compared to 24°C in *vti1-3* cells. There may be some partial complexes between Vam3p and Vam7p, which do not contain the other SNAREs. This idea would be supported by the observation that a higher fraction of Vam7p than Vti1p is immunoprecipitated (compare TCL and IP). On the other hand, this could also be explained by lower amounts of Vam7p present in the cell. The degree of coimmunoprecipitation is very low for Nyv1p, which may be due to incorporation of Ykt6p as alternative R-SNARE. These data indicate that SNARE complex formation was sufficient for transport to the vacuole with slower kinetics.

Tlg2p is a Qa-SNARE that forms a complex with the Qb-SNARE Vti1p, the Qc-SNARE Tlg1p and the R-SNARE Snc1p or Snc2p [8]. It is localized to the TGN, late Golgi and early endosomal structures. We constructed an N-terminal myc-tagged Tlg2p (myc-Tlg2p) to analyze complex assembly at the TGN/early endosome. Myc-Tlg2p was expressed from a *CEN*-based plasmid under control of its own regulatory element to produce endogenous levels. In wild type and *vti1-3* cells, Vti1p coprecipitated with myc-Tlg2p at 24°C (Fig. 6A). Again, reduced amounts of coimmunoprecipitated *vti1-3p* were observed in *vti1-3* cells at 37°C (13% ±2.6%, n = 3). Only small changes were observed for coimmunoprecipitation of Tlg1p. The reduced amounts of SNARE complexes may cause the observed GFP-Snc1p recycling defect in *vti1-3* cells at 37°C. It is worth noting that it is not possible to discriminate between *trans*-SNARE complexes bridging two membranes and *cis*-SNARE complexes on the same membrane with this coimmunoprecipitation approach. A SNARE complex locked in the *trans* state would not lead to fusion of a transport vesicle with its target membrane, but the SNARE complex partners would still coprecipitate.

**Figure 7:**
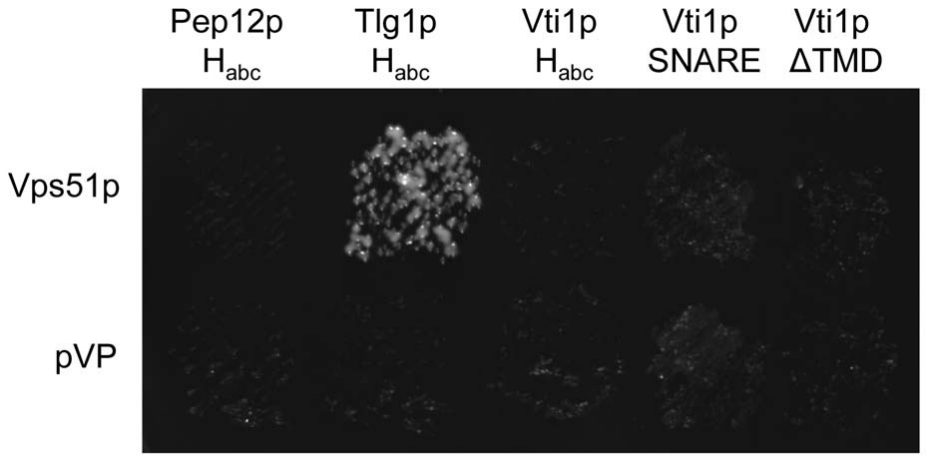
Vti1p H_abc_ domain did not interact with Vps51p. Two-hybrid interactions were detected by the ability of yeast cells (L40) to grow on selective plates. A fusion of the DNA-binding domain of LexA and the H_abc_ domain of Tlg1p (amino acids 1-137, pBK172) interacted with full-length Vps51p fused to the VP16 activation domain (pMG44). No interaction was detected for the H_abc_ domain of Vti1p (AA 1–115, pBK118), the H_abc_ domain of Pep12p (AA 1–200, pBK171), the SNARE motif of Vti1p (AA 116–188, pMG45) or Vti1p without its transmembrane domain (AA 1-188, pMG46). LexA fusion constructs with the VP16 alone (pVP) were used as negative controls.

To address the link between defective GFP-Snc1p recycling and reduced SNARE complex assembly at the TGN, we investigated a potential link between Vti1p and the GARP tethering complex. Vps51p, Vps52p, Vps53p and Vps54p are members of the GARP tethering complex which functions to tether vesicles derived from endosomes to the TGN [27]. GARP function is required for the retrieval of several recycling transmembrane proteins such as Kex2p, Vps10p and Snc1p to the TGN. In *S. cerevisiae*, Vps51p interacts directly with the H_abc_ domain of Tlg1p, thus mediating the association of the GARP complex with the SNARE machinery [17], [28]. In *C. elegans*, a direct interaction between VPS-51 and VTI-1 was recently observed [29]. This prompted us to check for an interaction of Vps51p with Vti1p with yeast-two-hybrid assays (Fig. 7). An interaction with Vps51p was observed for Tlg1p H_abc_ as expected demonstrating that the Vps51 two hybrid fusion protein was functional. However, no interaction was seen for Vps51 with Pep12p H_abc_ or Vti1p H_abc_ even though both fusion proteins are able to interact with Ent3p in two hybrid assays. In addition, Vps51p did not interact with the SNARE motif of Vti1p or a Vti1p construct lacking only its transmembrane domain (TMD) indicating that other domains of Vti1p do not facilitate complex formation. These data suggest that Vti1p does not interact with Vps51p in *S. cerevisiae*.

### Levels and Stability of Late Endosomal SNAREs

**Figure 8:**
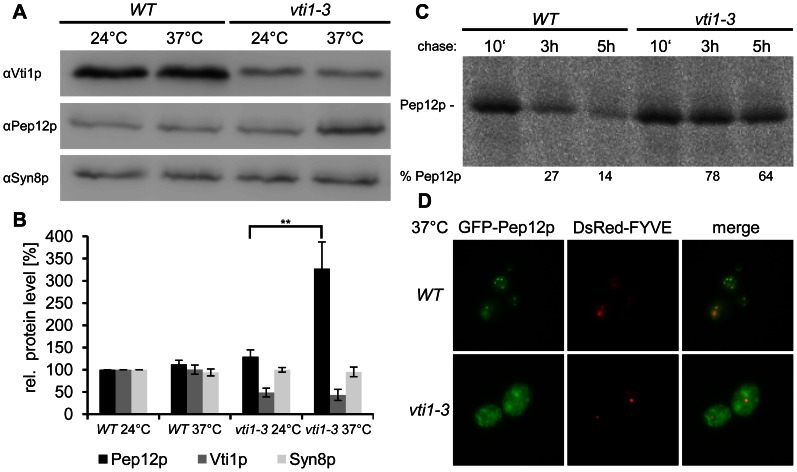
Levels and stability of late endosomal SNAREs. (A) Protein level of Pep12p was drastically increased in *vti1-3* cells after 2 h at 37°C. Protein extracts were analyzed by SDS-PAGE and immunoblotting. (B) Western blots were quantified and normalized to the amount in wild type cells at 24°C. Three independent experiments were quantified. mean ±standard deviation, **P<0.01, unpaired Student’s t test (C) Pep12p was more stable in *vti1-3* cells at 37°C. Cells were labeled with [^35^S]methionine and [^35^S]cysteine for 25 min and chased for 10 min, 3 h or 5 h. Pep12p was immunoprecipitated from the cellular extracts and the percentage of Pep12p remaining after 3 and 5 hours was calculated. Strains used: SEY6210 (WT), SCY14 (*vti1-3*). (D) GFP-Pep12p accumulated in intracelullar spots that did not colocalize with DsRed-FYVE in *vti1-3* cells after 2 h at 37°C. Strains used: SEY6210 pTK16 (WT GFP-Pep12p), SCY14 pTK16 (*vti1-3 *GFP-Pep12p).

The drastic effects on formation of the late endosomal SNARE complex in *vti1-3* cells may alter trafficking of these SNAREs leading to changes in stability. Therefore we investigated whether the *vti1-3* mutation altered the protein levels of late endosomal SNAREs and influenced their stability. Cells were grown to log-phase and remained at 24°C or were shifted to 37°C for 2 h. Protein extracts were prepared as described in the method section. The protein level of Pep12p was comparable to wild type at 24°C but significantly increased at 37°C in *vti1-3* cells (Fig. 8A, 8B). No change in protein level was detectable for Syn8p. The observed increased protein level of Pep12p in *vti1-3* cells at 37°C prompted us to analyze protein stability of Pep12p in more detail by pulse-chase labeling followed by immunoprecipitations after different chase periods. Stability of Pep12p was greatly increased in *vti1-3* cells at 37°C compared to wild type cells (Fig. 8C). After 3 h of chase, 36% (±13%, n = 2) of Pep12p was detectable in wild type cells, and 82% (±6%) in *vti1-3* cells. Pep12p stability was similarly affected after 5 h of chase (Pep12p in wild type: 23% ±12%, in *vti1-3* 64% ±0.5%). In wild type cells, GFP-Pep12p was localized in few intracellular spots that colocalized with DsRed-FYVE, a late endosomal marker. GFP signals in the lumen of many vacuoles suggest a vacuolar degradation of GFP-Pep12p. In contrast, GFP-Pep12p accumulated in small structures throughout the cell that did not colocalize with DsRed-FYVE in *vti1-3* cells after 2 h at 37°C (Fig. 8D).Taking into account that Vti1p does not coprecipitate with Pep12p in *vti1-3* cells at 37°C, these data suggest that a Vti1p - Pep12p interaction, possibly as part of a complete SNARE complex, is needed for efficient Pep12p degradation. However, we found no indication for changes in degradation of Syn8p, a SNARE complex partner of Pep12p.

### Secondary Structure of Mutant Vti1p H_abc_ Domains

**Figure 9:**
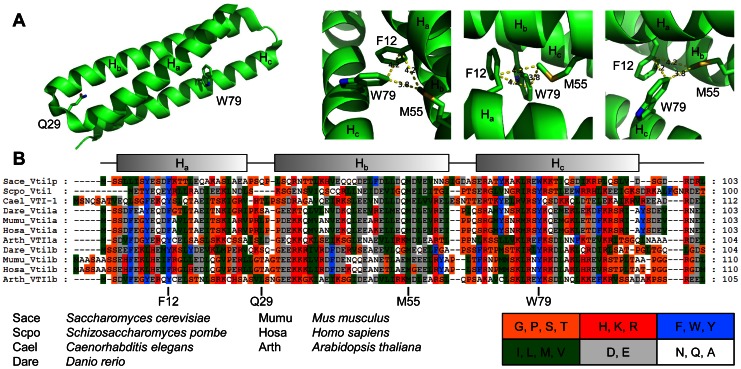
Position of Q29 and W79 in the crystal structure of Vti1p H_abc_ domain and their evolutionary conservation. (A) Left panel: Q29 is localized in the loop between helix H_a_ and helix H_b_. W79 is localized on helix H_c_, with its side chain turned towards the inner part of the three-helix-bundle, facing helix H_a_. Right panels: The crystal structure reveals a close proximity of W79 to F12 on helix H_a_ and to M55 on helix H_b_. These amino acids form part of the hydrophobic core of the three-helix-bundle. Data from PDB: 3ONJ; Distances in Å. (B) Vti1p F12, M55 and W79 are conserved across species while Vti1p Q29 is not conserved. Positions of α-helices in Vti1p are indicated above the alignment. Amino acids 1–120 of Vti1p from *Saccharomyces cerevisiae* and homologs from *Schizosaccharomyces pombe*, *Caenorhabditis elegans*, *Danio rerio*, *Mus musculus*, *Homo sapiens* and *Arabidopsis thaliana* were aligned using a PRALINE multiple sequence alignment (BLOSUM62 exchange weight matrix, associated gap penalties: 12 and 1. PSI-BLAST pre-profile processing (Homology-extended alignment), 3 iterations, E-value cut-off: 0.01, DSSP-defined secondary structure search, secondary structure prediction using PSIPRED).

**Figure 10:**
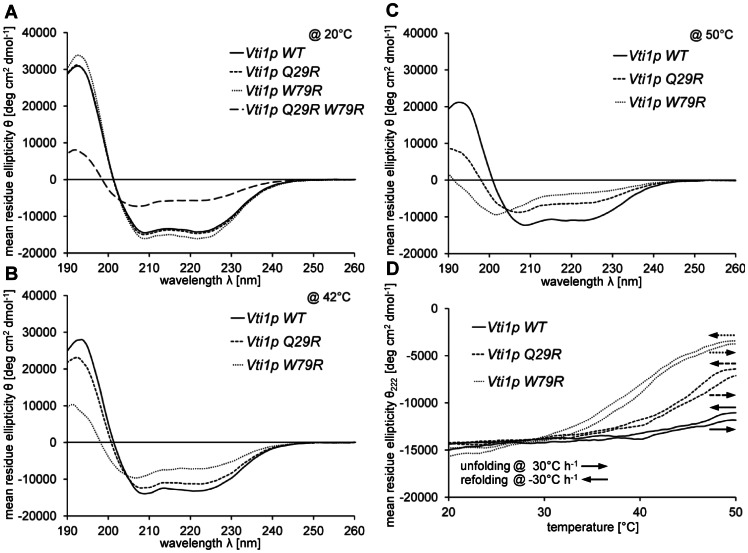
Analyses of secondary structure of wild type and mutant Vti1p H_abc_ by circular dichroism spectroscopy (CD). (A) CD spectra of Vti1p H_abc_ wild type (solid line), Q29R (short dashed line), W79R (dotted line) and Q29R W79R (long dashed line) at 20°C. The wild type and the mutants Q29R and W79R displayed high α-helical content, while the double mutant Q29R W79R was mostly unfolded. (B) CD spectra of Vti1p H_abc_ wild type (solid line), Q29R (short dashed line) and W79R (dotted line) at 42°C. Wild type and the mutant Q29R retained most of their α-helical content, while the mutant W79R was mostly unfolded. (C) CD spectra of Vti1p H_abc_ wild type (solid line), Q29R (short dashed line) and W79R (dotted line) at 50°C. The wild type retained some of its α-helical content, while the mutant Q29R was mostly unfolded and the mutant W79R was completely unfolded. (D) Unfolding of wild type and mutant Vti1p H_abc_ domain was reversible. Thermal un- and refolding was monitored by CD spectroscopy at 222 nm. Temperature was increased at a rate of 30°C h^−1^. Samples were kept at 50°C for 5 min before temperature was lowered at a rate of −30°C h^−1^.

To investigate the structural basis for the defects in *vti1-3p* the positions of the mutated residues Q29R and W79R were checked using the published crystal structure [21]. Q29 is part of the small loop between helix H_a_ and H_b_ (Fig. 9A). W79 is located in the middle of helix H_c_ far away from Q29 pointing towards the interior of the three-helix-bundle. Therefore it is unlikely that a ligand forms an interaction surface which includes both of these residues. It is more likely that these amino acid exchanges have a global impact on folding of this domain and the secondary structure. We studied the impact of the mutations on the secondary structure of the Vti1p H_abc_ domain by circular dichroism (CD) spectroscopy (Fig. 10A–D). The CD spectrum of purified recombinant wild type Vti1p H_abc_ recorded at 20°C suggests high α-helical content with a calculated α-helicity of 37%. This is comparable to earlier CD studies of the cytosolic part of Vti1p [30]. At 20°C, the CD spectra of the single mutants Q29R and W79R were comparable to wild type (38% and 42% α-helicity, respectively), while the α-helicity of the double mutant Q29R W79R was substantially decreased (15%, Fig. 10A). To evaluate the influence of the single mutants on secondary structure, we recorded CD spectra of wild type and the single mutants at elevated temperatures. At 42°C, wild type and the Q29R mutant displayed considerable α-helical content (34% and 29% α-helicity, respectively) while the α-helicity of the W79R mutant was reduced to 18% (Fig. 10B). At 50°C, only the wild type retained most of its α-helical content (28% α-helicity) while α-helicity was reduced to 16% and 9% in the Q29R and the W79R mutant, respectively (Fig. 10C). The isodichroic point at ∼204 nm suggests a transition from α-helical to random coil conformation [31]. These data indicate that the temperature-dependent phenotype of the *vti1-3* mutant is based on temperature-induced unfolding of the H_abc_ domain. As Golgi sorting recovered quickly and vacuolar sorting recovered slowly in *vti1-3* cells *in vivo* (Fig. 3B), we investigated whether unfolding of the H_abc_ domain was reversible *in vitro*. The CD spectra of proteins with α-helical secondary structure show characteristic minima at 208 nm and 222 nm. Increasing values of θ correlate with reduced α-helical content. We studied unfolding and refolding of wild type and mutant Vti1p H_abc_ domain by CD spectroscopy at 222 nm (Fig. 10D). Unfolding of wild type and mutant H_abc_ domain was almost fully reversible as indicated by similar values of θ before heating and after cooling. As suggested by the recorded CD spectra at 20°C, 42°C and 50°C (Fig. 10A–C), unfolding of Vti1p W79R was observed at lower temperatures than Vti1p Q29R and Vti1p Q29R unfolded at lower temperatures than Vti1p WT.

## Discussion

**Figure 11:**
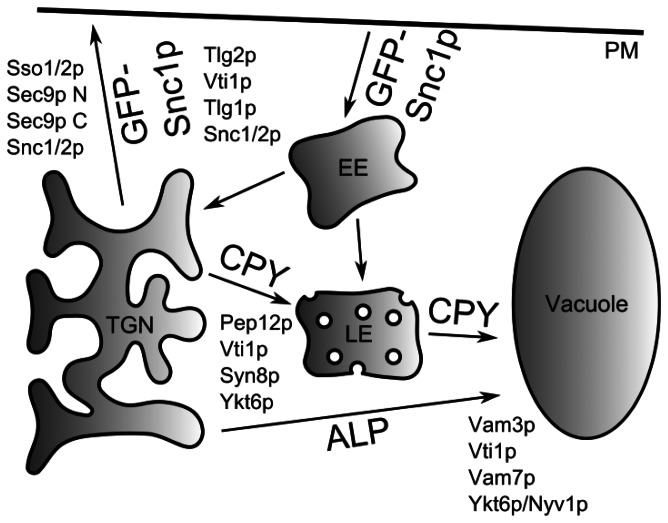
Vti1p containing SNARE complexes and their role in transport. A complex of Pep12p, Vti1p, Syn8p and Ykt6p is required for transport of CPY to the late endosome (LE). ALP is transported directly to the vacuole, which requires a complex consisting of Vam3p, Vti1p, Vam7p and Ykt6p or Nyv1p. GFP-Snc1p serves as a marker for recycling from the plasma membrane via early endosomes (EE) to the trans Golgi network (TGN). Tlg2p, Vti1p, Tlg1p and Snc1p or Snc2p function in this transport step.

Many SNARE proteins possess an autonomously folded N-terminal antiparallel three-helix bundle (H_abc_) domain in addition to their SNARE motif and C-terminal transmembrane domain [1]. In this study, we were able to demonstrate that a folded H_abc_ domain of the yeast Qb-SNARE Vti1p is required for anterograde traffic from the TGN to the LE and for retrograde traffic from early endosomes to the TGN but traffic to the vacuole is only delayed (Fig. 11).

A block in TGN to LE trafficking was observed in *vti1-3* cells, which secreted CPY with a rapid onset at 37°C. This allele carries two amino acid exchanges, Q29R and W79R, in the H_abc_ domain. Cells with the corresponding single amino acid exchange displayed no sorting defect. Analyses of the secondary structure of recombinantly expressed wild type and mutant Vti1p H_abc_ domain with CD spectroscopy revealed that α-helicity of the Q29R W79R mutant H_abc_ domain is drastically reduced at 20°C. However, our *in vivo* data on CPY sorting shows no defect at 24°C. A similar discrepancy between *in vivo* and *in vitro* data is seen for *vti1-1* and *vti1-2* mutants in homotypic vacuolar fusion. Although *vti1-1* and *vti1-2* cells show no defect in ALP trafficking at 24°C *in vivo* [32] and only *vti1-2* cells are affected slightly at 30°C [33], vacuoles isolated from *vti1-1* and *vti1-2* cells display severe fusion phenotypes at 27°C *in vitro* [6]. While the secondary structure was not affected at 20°C CD spectra at elevated temperatures indicate that the single amino acid exchange Q29R or W79R, respectively, destabilize the H_abc_ domain, but to a lesser extent than the double mutation. We conclude that these single amino acid exchanges alone do not sufficiently destabilize folding *in vivo*, as indicated by correct sorting of CPY at 37°C, but both mutations together result in a temperature-dependent defect due to unfolding of the H_abc_ domain. The mutation W79R destabilizes the protein more severely than the mutation Q29R. Q29 is localized in the turn between helix H_a_ and helix H_b_. W79 is localized on helix H_c_, with its side chain turned towards the inner part of the three-helix-bundle, facing helix H_a_ (Fig. 9A). Sequence alignment shows that W79 is conserved across species (Fig. 9B). The crystal structure reveals a close proximity to other hydrophobic amino acids, F12 on helix H_a_ and M55 on helix H_b_ (Fig. 9A), which are conserved as well (Fig. 9B). These amino acids form part of the hydrophobic core of the three-helix-bundle, which is needed for structural stability. Analyses of SNARE complex assembly revealed that the late endosomal SNARE complex failed to assemble in *vti1-3* cells at 37°C, indicating the requirement of a correctly folded Vti1p H_abc_ domain for SNARE complex assembly.

Recycling of GFP-Snc1p is defective in *vti1-3* cells at 37°C, indicating a block in early endosome to TGN traffic. While the TGN SNARE complex was still able to assemble, it was reduced in quantity. One possibility is that complex levels fell below a critical concentration to allow for efficient fusion. The copreciptiation experiment might have isolated a non-functional *trans* complex, which cannot undergo the conformational change to a *cis* complex during membrane fusion. This would lead to vesicles tethered to the TGN, unable to fuse with the target membrane. We expect that the observed defect in GFP-Snc1p recycling is directly due to defects in retrograde transport to the TGN and not caused indirectly by defects in TGN to late endosome transport. *vti1* mutants studies so far have very specific defects in certain transport steps. *vti1-1* cells display a complete temperature sensitive block in transport from the TGN to late endosomes [23]but transport to the vacuole and recycling of GFP-Snc1p are not affected [32], [33]. *vti1-11* cells are defective in transport from the TGN to late endosomes and in transport to the vacuole but GFP-Snc1p recycling is still functional. In addition, this retrograde transport step to the TGN is clearly separate from transport from the TGN to the late endosome because *pep12Δ* cells secret p2CPY completely [34], but do not show a GFP-Snc1p recycling defect [25].

A potential role of Vps51p, member of the GARP tethering complex, was investigated. In *C. elegans*, VPS-51 interacts with SYX-5, SYX-16 and VTI-1, but not with SYX-6 [29]. We could not detect an interaction between Vps51p and Vti1p. Interactions between GARP and SNARE in *C. elegans* appear to differ drastically from *S. cerevisiae*, because Vps51p interacts with Tlg1p (yeast SYX-6) [35], and not with Tlg2p (yeast SYX-16) [17] or Sed5p (yeast SYX-5) [36]. In addition, it was shown that human Vps51 (termed Ang2/fat-free) interacts with Syntaxin 6 [37]. This indicates that GARP - SNARE interactions may not be well conserved across all species.

The fast onset of the sorting defect of CPY points to a direct role of the Vti1p H_abc_ domain in fusion at the late endosome. The slow recovery of CPY transport to the vacuole might point to an irreversible unfolding of the protein, which then needs to be replaced by newly synthesized protein. However, the fast loss of the endoplasmatic reticulum (ER) form p1CPY upon return to 24°C indicates that refolding occurs *in vivo*. *In vitro* studies demonstrate that wild type Vti1p and wild type Vti1p H_abc_ as well as mutants with the amino acid exchange Q29R or W79R, respectively, are able to refold at least partially after heat denaturation ([30], Fig. 10D). The slow recovery might also be explained by the observed defect in transport from endosomes to the TGN resulting in defective GFP-Snc1p recycling. Vps10p functions as the CPY sorting receptor at the TGN. Vps10p with bound CPY is sorted into budding vesicles, which are destined to fuse with late endosomes. In late endosomes, CPY dissociates from its receptor and is transported to the vacuole while Vps10p requires retrieval to the TGN for continued sorting of CPY. Anterograde and retrograde traffic are disturbed concurrently in *vti1-3* cells upon shift to 37°C, severely impairing trafficking between TGN and the late endosome. Disturbed recycling of Vps10p may deplete Vps10p from the TGN, which would lead to a lag in restoring correct CPY sorting upon shift back to 24°C. In line with this hypothesis, a slow recovery of CPY sorting is observed in *vti1-2* cells [23] as well as a temperature-sensitive GFP-Snc1p recycling defect [33]. By contrast, *vti1-1* and *vti1-11* cells do not display a defect in retrograde transport to the TGN and CPY sorting recovers faster.

ALP was transported to the vacuole with slower kinetics in the *vti1-3* cells at 37°C but the extent of transport after 30 min chase reached almost wild type levels. By contrast, two mutant alleles with amino acid exchanges within the SNARE motif either transport ALP with wild type kinetics (*vti1-1*) or display a complete block in ALP traffic (*vti1-2*) [32]. The N-terminal domains of the vacuolar SNAREs Vam7p and Nyv1p are dispensable for *in vitro* fusion of vacuoles [38], [39], while vacuoles isolated from a Vam3p mutant lacking the H_abc_ domain fuse poorly and show a reduced recruitment of the vacuolar tethering complex HOPS to the *cis*-SNARE complex [40]. HOPS binds to the assembled vacuolar SNARE complex as well as to a complex comprised of vacuolar Q-SNAREs [41] and is needed for efficient fusion [42]. The SM protein Vps33p is part of the HOPS complex that binds Vam3p via its H_abc_ domain and SNARE motif [40], [43], [44]. Weakening the Vps33p-Vam3p interaction by mutations of either Vps33p or Vam3p leads to impaired fusion pore opening [45], probably by weakening binding of HOPS to the SNARE complex. In summary, HOPS recognizes and proof-reads assembled SNARE complexes, while the H_abc_ domains of the involved SNAREs have a regulatory function. A role of the Vti1p H_abc_ domain in rapid ALP trafficking underlines the importance of SNARE H_abc_ domains in efficient vacuolar transport *in vivo*.

GFP-Pep12p is degraded in the vacuole in wild type cells because GFP is observed in the vacuolar lumen. Analyses of late endosomal SNARE protein levels revealed an increased protein level of Pep12p in *vti1-3* cells at 37°C. A pulse-chase approach revealed a pronounced increase in stability of Pep12p in *vti1-3* cells at 37°C. Taken together with the observation that Vti1p does not coprecipitate with Pep12p in *vti1-3* cells at 37°C, this indicates a requirement for a Vti1p - Pep12p interaction, possibly as part of a complete SNARE complex, for efficient Pep12p degradation. An increased stability of Pep12p was also observed in *ent3*Δ cells [20]. Ent3p, an Epsin N-terminal homology domain (ENTH) protein functions in budding of clathrin coated vesicles. Ent3p interacts with the H_abc_ domains of Pep12p, Vti1p and Syn8p, functions as cargo adaptor for these SNAREs and mediates their correct localization *in vivo*. These data indicate that less Pep12p reaches the vacuole in *vti1-3* cells. Consistently, in *vti1-3* cells at 37°C, GFP-Pep12p accumulates in intracellular spots throughout the cell and does not colocalize with DsRed-FYVE (Fig. 8D). These accumulations probably represent GFP-Pep12p that is no longer able to reach the late endosome and vacuoles because fusion at the late endosome is not functional.We did not find evidence for changes in degradation of the Pep12p SNARE complex partner Syn8p. A resistance of Syn8p to vacuolar mislocalization has also been observed in *swf1*Δ cells [46]. Even though both Syn8p and Tlg1p require Swf1p for palmitoylation, only Tlg1p is mislocalized to the vacuole and degraded in *swf1*Δ cells, while a mislocalization of Syn8p to the vacuole was not observed. *vti1-3p* is degraded by the proteasome because it is stabilized by overexpression of ubiquitin K48R.

The SNARE motifs are mutated in other temperature-sensitive *vti1* mutants published so far (*vti1-1, vti1-2, vti1-11, vti1-12*) [47], as well as in temperature-sensitive *pep12* mutants (*pep12-60, pep12-61*) [48]. On a molecular level, the observed defects of these mutants are directly linked to the mutated amino acids, which are thought to destabilize the interacting layers of the SNARE complex. Interestingly, the N-terminal mutant *vti1-3*, despite its wild type SNARE motif, shows severe traffic defects. One well-known interaction partner of late endosomal SNAREs is Ent3p [20]. Ent5p is an AP180 N-terminal homology domain (ANTH) protein that has some functional redundancy to Ent3p, although it does not bind to endosomal SNAREs [20], [49]. *ent3Δ* cells display only a mild CPY sorting defect, which is aggravated to 83% mCPY in *ent3Δent5Δ* cells [19] even though Vti1p is mislocalized in *ent3Δ* and *ent3Δent5Δ* cells. However, compared to the CPY sorting defect of *vti1-3* cells, this defect is still very mild. We conclude that the phenotype of *vti1-3* cells cannot be explained solely with an abrogated interaction with Ent3p and with mislocalization of *vti1-3p*. An additional argument for a direct effect is the rapid onset of the CPY sorting defect in *vti1-3* cells after a shift to 37°C.

This study provides evidence for the importance of a folded H_abc_ domain in Vti1p. Our data indicate that the H_abc_ domain of Vti1p fulfills different roles in three different vesicle trafficking routes. In CPY trafficking, a folded H_abc_ domain was crucial for correct transport and for late endosomal SNARE complex assembly. A folded H_abc_ domain was needed for recycling of GFP-Snc1p. Unfolding of the H_abc_ domain had less drastic effects on assembly of the TGN SNARE complex. A folded H_abc_ domain increased transport kinetics of ALP to the vacuole. These differences are probably due to recruitment of different components of the membrane fusion machinery with an organelle specific localization.

## Materials and Methods

### Materials

Reagents were obtained from the following sources: enzymes for DNA manipulation from New England Biolabs (Beverly, MA, U.S.A), [^35^S]methionine and [^35^S]cysteine (EXPRE^35^S^35^S protein labeling mix) from Perkin Elmer (Waltham, MA, U.S.A), fixed Staphylacoccus aureus cells (Pansorbin) from Calbiochem (San Diego, CA), zymolyase from Seikagaku (Tokyo, Japan), Ni-NTA-Agarose from Qiagen (Hilden, Germany). All other reagents were purchased from Sigma (St Louis, MO, U.S.A), Serva (Heidelberg, Germany), Roth (Karlsruhe, Germany) or Merck (Darmstadt, Germany). Plasmid manipulations were performed in the E. coli strains DH5α or BL21CP(DE3) using standard media. Yeast strains were grown in rich medium (1% yeast extract, 1% peptone, 2% dextrose, YEPD) or standard minimal medium (SD) with appropriate supplements.

### Plasmids and Yeast Strains

Yeast strains and plasmids were constructed using standard genetic techniques. Yeast strains used in this study are described in Table 1. Plasmids used in this study are described in Table 2. Random mutagenesis of the *VTI1* gene and screening for mutants defective in CPY sorting is described [23]. A plasmid with five amino acid exchanges was identified. Subcloning resulting in pBK120 revealed that the amino acid exchanges Q29R W79R caused the phenotype. To integrate the *vti1-3* allele into the yeast genome, *vti1-3* DNA from pBK120 was subcloned into the integration vector pRS306 [50] to yield pSC9. SCY14 was constructed by integration of pSC9 linearized by *Bcl*I digestion into SEY6211, and looping out the wildtype *VTI1* on 5-FOA plates [51]. SSY4 was constructed by integration of pLO2010 linearized with *Eco*RI and looping out the wildtype *PEP4* on 5-FOA plates. SCY18 was constructed by integration of pTS15 linearized by *Eco*RI and *XhoI*.

**Table 1:** Yeast strains used in this study.

Strain	Genotype	Reference
SEY6210	*MATα leu2-3,112 ura3-52 his3-*Δ*200 trp1-*Δ*901 lys2-801 suc2-*Δ*9 mel-*	[58]
SEY6211	*MATa leu2-3,112 ura3-52 his3-*Δ*200 ade2-101 trp1-*Δ*901 suc2-*Δ*9 mel-*	[58]
SCY14	*MATa leu2-3,112 ura3-52 his3-*Δ*200 ade2-101 trp1-*Δ*901 suc2-*Δ*9 mel- vti1-3* (Q29R W79R)	this study
SSY4	*MATa leu2-3,112 ura3-52 his3-*Δ*200 ade2-101 trp1-*Δ*901 suc2-*Δ*9 mel- pep4*Δ	this study
BKY12	*MATα leu2-3,112 ura3-52 his3-*Δ*200 trp1-*Δ*901 lys2-801 suc2-*Δ*9 mel- pep4*Δ*::URA3*	[61]
SCY18	*MATa leu2-3,112 ura3-52 his3-*Δ*200 ade2-101 trp1-*Δ*901 suc2-*Δ*9 mel- pep4*Δ*::URA3 vti1-3*	this study
FvMY6	*MATα leu2-3,112 ura3-52 trp1 -*Δ*901 ade2-101 lys2-801 suc2-*Δ*9 mel- vti1*Δ*::HIS3*	[23]
L40	*MATa leu2-3,112 his3-*Δ*200 ade2-101 trp1-*Δ*901 LYS2::(lexAop)4-HIS3 URA3::(lexAop)4-lacZ gal80*	[57]

**Table 2:** Plasmids used in this study.

Plasmid	Description	Reference
pFvM29	1.8 kb containing *VTI1* in pRS316 (*CEN, URA3*)	[23]
pBK120	*vti1-3* in pRS314 (*CEN, TRP1*)	this study
pSC14	*vti1-3* in yEP351 (*2µ, LEU2*)	this study
pBK123	Vti1p Q29R in pRS316 (*CEN, URA3*)	this study
pBK128	Vti1p W79R in pRS316 (*CEN, URA3*)	this study
pSC9	*vti1-3* in pRS306 (*URA3*)	this study
pJeJe6	myc-Tlg2p in pRS316 (*CEN, URA3*)	this study
pMG50	myc-Tlg2p in pRS314 (*CEN, TRP1*)	this study
pGS416	GFP-Snc1p (*CEN, URA3*)	[25]
pJJ8	GFP-Snc1p in pRS314 (*CEN, TRP1*)	this study
pMG1	Vti1p WT AA1-115 in pET28b	[21]
pMG2	Vti1p Q29R W79R AA1-115 in pET28b	this study
pSP3	Vti1p Q29R AA1-115 in pET28b	this study
pSP4	Vti1p W79R AA1-115 in pET28b	this study
pTK16	GFP-Pep12p in pRS314 (*CEN, TRP1*)	this study
pTPQ127	DsRed-FYVE	[26]
pTPQ128	DsRed-Sec7p	[26]
pUB203	Ubiquitin K48R (*2µ, TRP1*)	[62]
pLexN	LexA DNA-binding domain for Y2H (*2μ, TRP1*)	[57]
pVP16-3	VP16 transactivation domain for Y2H (*2μ, LEU2*)	[57]
pMG44	Vps51p full-length in pVP16-3	this study
pBK171	Pep12p Habc (AA1-200) in pLexN	[20]
pBK172	Tlg1p Habc (AA1-137) in pLexN	[20]
pBK118	Vti1p Habc (AA1-115) in pLexN	[19]
pMG45	Vti1p SNARE (AA116-188) in pLexN	this study
pMG46	Vti1p ΔTMD (AA1-188) in pLexN	this study
pFW6	Syn8p AA1-169 in pET28b	[20]
pFvM140	Vam3p AA1-234 in pGEX5-1	this study
pTS15	*PEP4* disruption construct	[59]
pLO2010	*PEP4* disruption plasmid (loop-in:loop-out)	[60]

### Immunoprecipitation of ^35^S-labeled Proteins

Yeast cells were grown to log-phase at 24°C and 0.5 OD were preincubated at the indicated temperature for 15 min for temperature shift experiments. Cells were labeled with [^35^S]methionine and [^35^S]cysteine (70 µCi, 0.5 OD for CPY and 100 µCi, 0.5 OD for ALP) for 10 min and chased for 30 min at this temperature (if not stated otherwise). CPY was immunoprecipitated with a rabbit antiserum from cellular extracts (I) and medium (E), whereas ALP was only isolated from cellular extracts. For Vti1p and Pep12p stability assays, the cells were labeled for 25 minutes (350 µCi, 1.6 OD), chased for the indicated times and immunoprecipitated from cellular extracts (I) as described previously [32], [52], [53]. *vti1-3* cells carrying a plasmid encoding ubiquitin K48R (pUB203) under control of the *CUP1* promotor were grown to log-phase at 24°C in the presence of 100 µM CuSO_4_. CPY and ALP antisera were kindly provided by T.H. Stevens (University of Oregon). Immunoprecipitates were analysed by SDS-PAGE and autoradiography. A BAS-1800 II (Fuji) phosphor imager was used for quantification.

### Fluorescence Microscopy

For analyses of GFP–Snc1p (pJJ8) and DsRed-Sec7p (pTPQ128) localization, yeast cells were grown to early log-phase in appropriate SD minimal medium at 24°C, and then shifted to 37°C for 30 min. Cells were pelleted, resuspended in PBS and immediately viewed under a fluorescence microscope (Leica DM-5000 B with a Leica DFC350 FX CCD camera) as described [33]. For quantification of plasma membrane staining, at least 200 cells for each experiment were counted. For analyses of GFP-Pep12p (pTK16) and DsRed-FYVE (pTPQ127) localization, yeast cells were grown to log-phase in appropriate SD minimal medium at 24°C, and then shifted to 37°C for 2 h. Cells were pelleted, resuspended in PBS and immediately viewed under a fluorescence microscope as described. For analyses of Vti1p and Vps45p-3HA localization, indirect immunofluorescence was performed as described previously (Raymond et al. 1992) using an affinity-purified antiserum against Vti1p and a monoclonal antibody against the HA epitope (16B12, Covance). Primary antibodies were detected using appropriate Cy2- or Cy3- conjugated secondary antibodies (Jackson ImmunoResearch). Cells were grown to log-phase in appropriate SD minimal medium at 24°C, and then shifted to 37°C for 30 min. Cells were fixed with 3.7% formaldehyde for 30 min. Cells were harvested by centrifugation and resuspended in 4% paraformaldehyde 100 mM potassium phosphate pH 6.5 and incubated over night at room temperature. Cells were spheroplasted and permeabilized with 1% SDS/1M Sorbitol.

### Analyses of SNARE Complex Assembly by Coimmunoprecipitation

Immunoprecipitations of SNAREs were performed similar to published procedures [54]. Antiserum against Pep12p or Vam3p or a monoclonal antibody against c-myc (9E10) as well as pre-immune serum was cross-linked to protein G-sepharose with dimethylpimelimidate. 20 OD of cells (SSY4 and SCY18, with or without transformed pMG50) grown to log-phase were harvested and then spheroplasted for 1 h at 24°C in spheroplast buffer (1.2 M sorbitol, 50 mM K_2_HPO_4_ pH 7.3, 1 mM MgCl_2_) containing 300 µg/ml zymolyase. Spheroplasts were washed, resuspended in 5 ml YEPD/1 M sorbitol and incubated for 1 h at 24°C or 37°C. Cells were suspended in 1 ml of lysis buffer (20 mM HEPES-KOH pH 7.0, 100 mM KCl, 2 mM EDTA, 0.5% Triton X-100+ protease inhibitors) and dounced 20 times on ice. The detergent extract was centrifuged for 20 min at 200.000 *g*. 750 µL of supernatant and 45 µL of beads were incubated overnight at 4°C. Beads were washed five times with lysis buffer and suspended in 50 µL of 1x sample buffer without β-mercaptoethanol. Samples were analyzed by SDS-PAGE and immunoblotting. Antisera against Tlg1p were kindly provided by HRB Pelham, MRC Cambridge [7] and against Nyv1p and Vam7p by C Ungermann, Universität Osnabrück [55].

### Production of Syn8p and Vam3p Antisera

Anti-Syn8p rabbit serum was raised against 6His-Syn8p (N-terminal domain, residues 1–169). The fusion protein was expressed in *E. coli* and purified using Ni-NTA-agarose. Anti-Syn8p serum recognized a protein with the expected molecular mass in wild type but not in *syn8Δ* cell extracts. Anti-Vam3p rabbit serum was raised against GST-Vam3p (soluble domain, residues 1-264) expressed in *E. coli* and purified using glutathione-agarose. Anti-Vam3p serum recognized a protein with the expected molecular mass in wild type but not in *vam3Δ* cell extracts.

### Analyses of SNARE Protein Levels

For yeast protein extract preparation, cells were grown at 24°C to log-phase. For temperature-dependent experiments, cells grown at 24°C were shifted to 37°C for 2 h. Cells were harvested by centrifugation, washed once with water, and the pellet was resuspended in 40 µL/OD of lysis buffer (8 M urea, 5% SDS, 50 mM Tris-HCl pH 6.8, 5% β-mercaptoethanol+protease inhibitors) preheated to 70°C. Glass beads were added and cells were lysed by vigorous shaking using a vortexer. The mixture was incubated for 10 min at 70°C and centrifuged for 5 min at 17,000 *g*. Equal volumes (corresponding to equal ODs) of protein extracts were subjected to SDS-PAGE and proteins were transferred onto a nitrocellulose membrane. Protein amounts and transfer were controlled by ponceau S staining. Rabbit antisera were used for the detection of Vti1p, Pep12p, and Syn8p. An incubation with HRP-conjugated secondary antibodies was followed by visualization with ECL reagents using a CCD camera (Fujifilm LAS 3000).

### Analyses of Secondary Structure with Circular Dichroism

Wildtype and mutant Vti1p H_abc_ domain were recombinantly expressed with a N-terminal 6His tag in the *E. coli* strain BL21CP(DE3) using the plasmid pET28b(+) and purified by metal affinity chromatography using Ni-NTA agarose (Qiagen, Hilden, Germany). The purified recombinant proteins were analyzed by SDS-PAGE and Coomassie Blue staining for purity. Purified protein was dialyzed against 10 mM NaH_2_PO_4_ 100 mM Na_2_SO_4_.4 and analyzed by CD spectroscopy at a concentration of 0.2 mg/mL as determined by Bradford assay. CD spectra were recorded on a Jasco J-810 spectropolarimeter. Recorded spectra were smoothed with a Savitsky-Golay function and mean residue ellipticity was calculated. The α-helical content was calculated from ellipticity using the program DichroProt [56]. Thermal un- and refolding of wild type and mutant Vti1p H_abc_ was monitored by CD spectroscopy at 222 nm. Temperature was increased at a rate of 30°C h^−1^. Samples were kept at 50°C for 5 min before temperature was lowered at a rate of −30°C h^−1^.

### Yeast Two Hybrid

Yeast two-hybrid assays were performed as described [57] in L40 cells. The H_abc_ domain of Vti1p (AA1-115) [19], the SNARE motif of Vti1p (AA116-188), Vti1p without its transmembrane domain (AA 1-188), the H_abc_ domain of Pep12p (AA 1-200) and the H_abc_ domain of Tlg1p (AA 1-137) [20] in pLexN were used as bait vectors. Vps51p in pVP16-3 was used as prey vector. Yeast cells containing pairs of LexA DNA-binding domains and VP16 activation domain fusions were streaked out on plates with minimal medium lacking uracil, lysine, tryptophan, leucine and histidine, which was complemented with 20 mM 3-aminotriazole to study specific two-hybrid interactions.

## Supporting Information

Figure S1 **Localization of Vti1p,** ***vti1-3p*** **and Vps45p-3HA in wild-type and** ***vti1-3*** **cells.**
(PDF)

Figure S2 **GFP-Snc1p and DsRed-Sec7p localization at 24°C and 37°C in wild type and** ***vti1-3*** **cells.**
(PDF)

## References

[1] Jahn R, Scheller RH (2006) SNAREs - engines for membrane fusion. Nat Rev Mol Cell Biol 7: 631–643. 16912714 10.1038/nrm2002

[2] Kloepper TH, Kienle CN, Fasshauer D (2007) An elaborate classification of SNARE proteins sheds light on the conservation of the eukaryotic endomembrane system. Mol Biol Cell 18: 3463–3471. 17596510 10.1091/mbc.E07-03-0193 PMC1951749

[3] Dilcher M, Köhler B, Fischer von Mollard G (2001) Genetic interactions with the yeast Q-SNARE VTI1 reveal novel functions for the R-SNARE YKT6. J Biol Chem 276: 34537–34544. 11445562 10.1074/jbc.M101551200

[4] Kweon Y, Rothe A, Conibear E, Stevens TH (2003) Ykt6p is a multifunctional yeast R-SNARE that is required for multiple membrane transport pathways to the vacuole. Mol Biol Cell 14: 1868–1881. 12802061 10.1091/mbc.E02-10-0687 PMC165083

[5] Lewis MJ, Pelham HRB (2002) A new yeast endosomal SNARE related to mammalian syntaxin 8. Traffic 3: 922–929. 12453154 10.1034/j.1600-0854.2002.31207.x

[6] Ungermann C, Fischer von Mollard G, Jensen ON, Margolis N, Stevens TH, et al (1999) Three v-SNAREs and two t-SNAREs, present in a pentameric cis-SNARE complex on isolated vacuoles, are essential for homotypic fusion. J Cell Biol 145: 1435–1442. 10385523 10.1083/jcb.145.7.1435 PMC2133161

[7] Holthuis JCM, Nichols BJ, Dhruvakumar S, Pelham HRB (1998) Two syntaxin homologues in the TGN/endosomal system in yeast. Embo J 17: 113–126. 9427746 10.1093/emboj/17.1.113 PMC1170363

[8] Brickner J, Blanchette J, Sipos G, Fuller R (2001) The Tlg SNARE complex is required for TGN homotypic fusion. J Cell Biol 155: 969–978. 11739408 10.1083/jcb.200104093 PMC2150899

[9] Banfield DK, Lewis MJ, Pelham HR (1995) A SNARE-like protein required for traffic through the Golgi complex. Nature 375: 806–809. 7596416 10.1038/375806a0

[10] Lupashin VV, Pokrovskaya ID, McNew JA, Waters MG (1997) Characterization of a novel yeast SNARE protein implicated in Golgi retrograde traffic. Mol Biol Cell 8: 2659–2676. 9398683 10.1091/mbc.8.12.2659 PMC25735

[11] McNew JA, Søgaard M, Lampen NM, Machida S, Ye RR, et al (1997) Ykt6p, a prenylated SNARE essential for Endoplasmic Reticulum-Golgi transport. J Biol Chem 272: 17776–17783. 9211930 10.1074/jbc.272.28.17776

[12] Toonen RFG, Verhage M (2003) Vesicle trafficking: pleasure and pain from SM genes. Trends Cell Biol 13: 177–186. 12667755 10.1016/s0962-8924(03)00031-x

[13] Yamaguchi T, Dulubova I, Min SW, Chen XH, Rizo J, et al (2002) Sly1 binds to Golgi and ER syntaxins via a conserved N-terminal peptide motif. Dev Cell 2: 295–305. 11879635 10.1016/s1534-5807(02)00125-9

[14] Dulubova I, Yamaguchi T, Gao Y, Min SW, Huryeva I, et al (2002) How Tlg2p/syntaxin 16 'snares' Vps45. Embo J 21: 3620–3631. 12110575 10.1093/emboj/cdf381 PMC126126

[15] Ren Y, Yip CK, Tripathi A, Huie D, Jeffrey PD, et al (2009) A Structure-Based Mechanism for Vesicle Capture by the Multisubunit Tethering Complex Dsl1. Cell 139: 1119–1129. 20005805 10.1016/j.cell.2009.11.002 PMC2806190

[16] Meiringer CTA, Rethmeier R, Auffarth K, Wilson J, Perz A, et al (2011) The Dsl1 Protein Tethering Complex Is a Resident Endoplasmic Reticulum Complex, Which Interacts with Five Soluble NSF (N-Ethylmaleimide-sensitive Factor) Attachment Protein Receptors (SNAREs) - implications for fusion and fusion regulation. J Biol Chem 286: 25039–25046. 21550981 10.1074/jbc.M110.215327 PMC3137077

[17] Conibear E, Cleck JN, Stevens TH (2003) Vps51p mediates the association of the GARP (Vps52/53/54) complex with the late Golgi t-SNARE Tlg1p. Mol Biol Cell 14: 1610–1623. 12686613 10.1091/mbc.E02-10-0654 PMC153126

[18] Antonin W, Dulubova I, Arac D, Pabst S, Plitzner J, et al (2002) The N-terminal domains of syntaxin 7 and vti1b form three-helix bundles that differ in their ability to regulate SNARE complex assembly. J Biol Chem 277: 36449–36456. 12114520 10.1074/jbc.M204369200

[19] Chidambaram S, Mullers N, Wiederhold K, Haucke V, von Mollard GF (2004) Specific interaction between SNARES and epsin N-terminal homology (ENTH) domains of epsin-related proteins in trans-Golgi network to endosome transport. J Biol Chem 279: 4175–4179. 14630930 10.1074/jbc.M308667200

[20] Chidambaram S, Zimmermann J, von Mollard GF (2008) ENTH domain proteins are cargo adaptors for multiple SNARE proteins at the TGN endosome. J Cell Sci 121: 329–338. 18198191 10.1242/jcs.012708

[21] Wang J, Gossing M, Fang PF, Zimmermann J, Li X, et al (2011) Epsin N-terminal homology domains bind on opposite sides of two SNAREs. Proc Nat Acad Sci 108: 12277–12282. 21746902 10.1073/pnas.1013101108 PMC3145707

[22] Conibear E, Stevens TH (1998) Multiple sorting pathways between the late Golgi and the vacuole in yeast. Biochem Biophys Acta 1404: 211–230. 9714809 10.1016/s0167-4889(98)00058-5

[23] Fischer von Mollard G, Nothwehr SF, Stevens TH (1997) The yeast v-SNARE Vti1p mediates two vesicle transport pathways through interactions with the t-SNAREs Sed5p and Pep12p. J Cell Biol 137: 1511–1524. 9199167 10.1083/jcb.137.7.1511 PMC2137825

[24] Protopopov V, Govindan B, Novick P, Gerst JE (1993) Homologs of the synaptobrevin/VAMP family of synaptic vesicle proteins function on the late secretory pathway in S. cerevisiae. Cell 74: 855–861. 8374953 10.1016/0092-8674(93)90465-3

[25] Lewis MJ, Nichols BJ, Prescianotto-Baschong C, Riezman H, Pelham HRB (2000) Specific retrieval of the exocytic SNARE Snc1p from early yeast endosomes. Mol Biol Cell 11: 23–38. 10637288 10.1091/mbc.11.1.23 PMC14754

[26] Proszynski TJ, Klemm RW, Gravert M, Hsu PP, Gloor Y, et al (2005) A genome-wide visual screen reveals a role for sphingolipids and ergosterol in cell surface delivery in yeast. Proc Nat Acad Sci 102: 17981–17986. 16330752 10.1073/pnas.0509107102 PMC1312417

[27] Conibear E, Stevens T (2000) Vps52p, Vps53p, and Vps54p form a novel multisubunit complex required for protein sorting at the yeast late Golgi. Mol Biol Cell 11: 305–323. 10637310 10.1091/mbc.11.1.305 PMC14776

[28] Fridmann-Sirkis Y, Kent HM, Lewis MJ, Evans PR, Pelham HRB (2006) Structural analysis of the interaction between the SNARE Tlg1 and Vps51. Traffic 7: 182–190. 16420526 10.1111/j.1600-0854.2005.00374.x

[29] Luo L, Hannemann M, Koenig S, Hegermann J, Ailion M, et al (2011) The Caenorhabditis elegans GARP complex contains the conserved Vps51 subunit and is required to maintain lysosomal morphology. Mol Biol Cell 22: 2564–2578. 21613545 10.1091/mbc.E10-06-0493 PMC3135481

[30] Tishgarten T, Yin FF, Faucher KM, Dluhy RA, Grant TR, et al (1999) Structures of yeast vesicle trafficking proteins. Prot Sci 8: 2465–2473. 10.1110/ps.8.11.2465 PMC2144180 10595551

[31] Holtzer ME, Holtzer A (1992) Alpha-helix to random coil transitions: determination of peptide concentration from the CD at the isodichroic point. Biopolymers 32: 1675–1677. 1472650 10.1002/bip.360321209

[32] Fischer von Mollard G, Stevens TH (1999) The Saccharomyces cerevisiae v-SNARE Vti1p is required for multiple membrane transport pathways to the vacuole. Mol Biol Cell 10: 1719–1732. 10359592 10.1091/mbc.10.6.1719 PMC25363

[33] Stein IS, Gottfried A, Zimmermann J, Von Mollard GF (2009) TVP23 interacts genetically with the yeast SNARE VTI1 and functions in retrograde transport from the early endosome to the late Golgi. Biochem J 419: 229–236. 19076069 10.1042/BJ20081973

[34] Becherer KA, Rieder SE, Emr SD, Jones EW (1996) Novel syntaxin homolog, Pep12p, required for the sorting of lumenal hydrolases to the lysosome-like Vacuole in yeast. Mol Biol Cell 7: 579–594. 8730101 10.1091/mbc.7.4.579 PMC275911

[35] Siniossoglou S, Pelham HRB (2002) Vps51p links the VFT complex to the SNARE Tlg1p. J Biol Chem 277: 48318–48324. 12377769 10.1074/jbc.M209428200

[36] Reggiori F, Wang CW, Stromhaug PE, Shintani T, Klionsky DJ (2003) Vps51 is part of the yeast Vps fifty-three tethering complex essential for retrograde traffic from the early endosome and Cvt vesicle completion. J Biol Chem 278: 5009–5020. 12446664 10.1074/jbc.M210436200 PMC1705970

[37] Perez-Victoria FJ, Schindler C, Magadan JG, Mardones GA, Delevoye C, et al (2010) Ang2/Fat-Free Is a Conserved Subunit of the Golgi-associated Retrograde Protein Complex. Mol Biol Cell 21: 3386–3395. 20685960 10.1091/mbc.E10-05-0392 PMC2947474

[38] Starai VJ, Jun Y, Wickner W (2007) Excess vacuolar SNAREs drive lysis and Rab bypass fusion. Proc Nat Acad Sci 104: 13551–13558. 17699614 10.1073/pnas.0704741104 PMC1959418

[39] Jun Y, Xu H, Thorngren N, Wickner W (2007) Sec18p and Vam7p remodel trans-SNARE complexes to permit a lipid-anchored R-SNARE to support yeast vacuole fusion. Embo J 26: 4935–4945. 18007597 10.1038/sj.emboj.7601915 PMC2140102

[40] Laage R, Ungermann C (2001) The N-terminal domain of the t-SNARE Vam3p coordinates priming and docking in yeast vacuole fusion. Mol Biol Cell 12: 3375–3385. 11694574 10.1091/mbc.12.11.3375 PMC60262

[41] Kramer L, Ungermann C (2011) HOPS drives vacuole fusion by binding the vacuolar SNARE complex and the Vam7 PX domain via two distinct sites. Mol Biol Cell 22: 2601–2611. 21613544 10.1091/mbc.E11-02-0104 PMC3135484

[42] Mima J, Hickey CM, Xu H, Jun Y, Wickner W (2008) Reconstituted membrane fusion requires regulatory lipids, SNAREs and synergistic SNARE chaperones. Embo J 27: 2031–2042. 18650938 10.1038/emboj.2008.139 PMC2516887

[43] Sato TK, Rehling P, Peterson MR, Emr SD (2000) Class C Vps protein complex regulates vacuolar SNARE pairing and is required for vesicle docking/fusion. Mol Cell 6: 661–671. 11030345 10.1016/s1097-2765(00)00064-2

[44] Dulubova I, Yamaguchi T, Wang Y, Sudhof TC, Rizo J (2001) Vam3p structure reveals conserved and divergent properties of syntaxins. Nat Struct Biol 8: 258–264. 11224573 10.1038/85012

[45] Pieren M, Schmidt A, Mayer A (2010) The SM protein Vps33 and the t-SNARE H-abc domain promote fusion pore opening. Nat Struct Mol Biol 17: 710–717. 20453860 10.1038/nsmb.1809

[46] Valdez-Taubas J, Pelham H (2005) Swf1-dependent palmitoylation of the SNARE Tlg1 prevents its ubiquitination and degradation. Embo J 24: 2524–2532. 15973437 10.1038/sj.emboj.7600724 PMC1176453

[47] Fischer von Mollard G, Stevens TH (1998) A Human homolog can functionally replace the yeast v-SNARE Vti1p in two vesicle transport pathways. J Biol Chem 273: 2624–2630. 9446565 10.1074/jbc.273.5.2624

[48] Burd CG, Peterson M, Cowles CR, Emr SD (1997) A novel Sec18p/NSF-dependent complex required for Golgi-to-endosome transport in yeast. Mol Biol Cell 8: 1089–1104. 9201718 10.1091/mbc.8.6.1089 PMC305716

[49] Duncan M, Costaguta G, Payne G (2003) Yeast epsin-related proteins required for Golgi-endosome traffic define a gamma-adaptin ear-binding motif. Nat Cell Biol 5: 77–81. 12483220 10.1038/ncb901

[50] Sikorski RS, Hieter P (1989) A system of shuttle vectors and yeast host strains designed for efficient manipulation of DNA in *Saccharomyces cerevisiae* . Genetics 122: 19–27. 2659436 10.1093/genetics/122.1.19 PMC1203683

[51] Boeke JD, LaCroute F, Fink GR (1984) A positive selection for mutants lacking orotidine-5′-phospahte decarboxylase activity in yeast:5-fluororotic acid resistance. Mol Gen Genet 197. 10.1007/BF00330984 6394957

[52] Vater CA, Raymond CK, Ekena K, Howald S, I., Stevens TH (1992) The *VPS1* protein, a homolog of dynamin required for vacuolar protein sorting in *Saccharomyces cerevisiae*, is a GTPase with two functionally separable domains. J Cell Biol 119: 773–786. 1429836 10.1083/jcb.119.4.773 PMC2289700

[53] Nothwehr SF, Roberts CJ, Stevens TH (1993) Membrane protein retention in the yeast Golgi apparatus: dipeptidyl aminopeptidase A is retained by a cytoplasmic signal containing aromatic residues. J Cell Biol 121: 1197–1209. 8509444 10.1083/jcb.121.6.1197 PMC2119699

[54] Lewis MJ, Rayner JC, Pelham HRB (1997) A novel SNARE complex implicated in vesicle fusion with the endoplasmic reticulum. Embo J 16: 3017–3024. 9214619 10.1093/emboj/16.11.3017 PMC1169920

[55] Ungermann C, Sato K, Wickner W (1998) Defining the functions of trans-SNARE pairs. Nature 396: 543–548. 9859990 10.1038/25069

[56] Deleage G, Geourjon C (1993) An interactive graphic program for calculating the secondary structure-content of proteins from circular-dichroism spctrum. Comput Appl Biosci 9: 197–199. 8481823 10.1093/bioinformatics/9.2.197

[57] Vojtek A, Hollenberg S (1995) Ras-Raf interaction: two-hybrid analysis. Methods Enzymol 255: 331–342. 8524119 10.1016/s0076-6879(95)55036-4

[58] Robinson JS, Klionsky DJ, Banta LM, Emr SD (1988) Protein sorting in *Saccharomyces cerevisiae*: isolation of mutants defective in the delivery and processing of multiple vacuolar hydrolases. Mol Cell Biol 8: 4936–4948. 3062374 10.1128/mcb.8.11.4936-4948.1988 PMC365587

[59] Ammerer G, Hunter CP, Rothman JH, Saari GC, Valls LA, et al (1986) *PEP4* gene of *Saccharomyces cerevisiae* encodes proteinase A, a vacuolar enzyme required for processing of vacuolar precursors. Mol Cell Biol 6: 2490–2499. 3023936 10.1128/mcb.6.7.2490-2499.1986 PMC367803

[60] Nothwehr SF, Conibear E, Stevens TH (1995) Golgi and vacuolar membrane proteins reach the vacuole in *vps1* mutant yeast cells via the plasma membrane. J Cell Biol 129: 35–46. 7698993 10.1083/jcb.129.1.35 PMC2120360

[61] Dilcher M, Veith B, Chidambaram S, Hartmann E, Schmitt HD, et al (2003) Use1p is a yeast SNARE protein required for retrograde traffic to the ER. EMBO J 22: 3664–3674. 12853481 10.1093/emboj/cdg339 PMC165609

[62] Finley D, Sadis S, Monia BP, Boucher P, Ecker DJ, et al (1994) Inhibition of proteolysis and cell-cycle progression in a multiubiquitination-deficient yeast mutant. Mol Cell Biol 14: 5501–5509. 8035826 10.1128/mcb.14.8.5501-5509.1994 PMC359070

