# Supplementary material for: Importance of the N-Terminal Domain of the Qb-SNARE Vti1p for Different Membrane Transport Steps in the Yeast Endosomal System
Source: PLoS One. 2013 Jun 12;8(6):e66304. doi: 10.1371/journal.pone.0066304 (PMC3680383; doi:10.1371/journal.pone.0066304)
Supplement: Figure S1 — Localization of Vti1p, vti1-3p and Vps45p-3HA in wild-type and vti1-3 cells. (PDF) [file pone.0066304.s001.pdf]

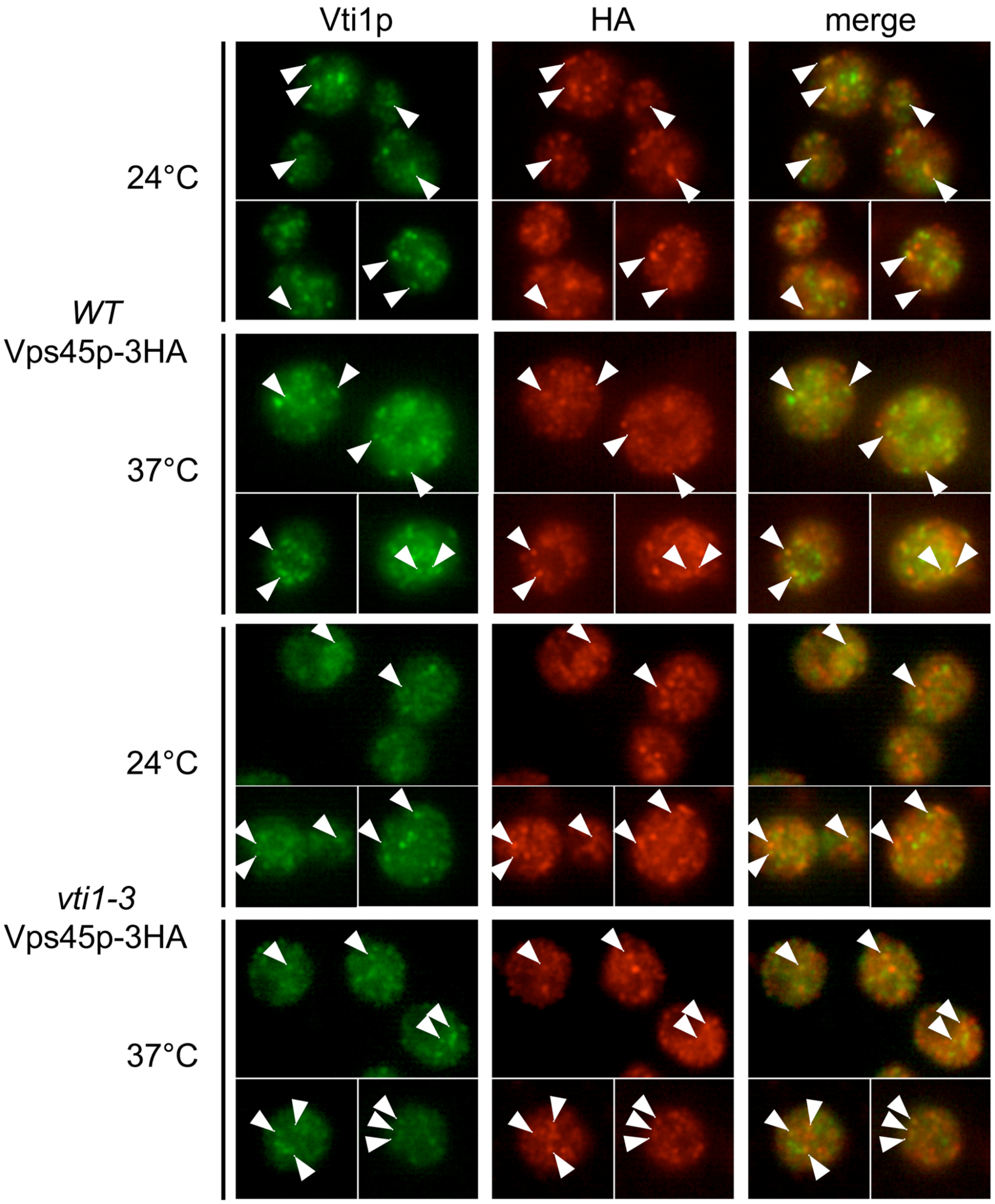

Figure S1 Gossing et al.

Figure S1. Localization of Vti1p, *vti1-3p* and Vps45p-3HA in wild-type and *vti1-3* cells. Vti1p and *vti1-3p* were localized in punctuate structures that colocalized with Vps45p-3HA in wild type and *vti1-3* cells at 24°C and 37°C. *vti1-3p* staining was less intense and slightly more diffuse compared to wild-type. Cells were grown to log-phase at 24°C and then shifted to 37°C for 30 min. Indirect immunofluorescence was performed using an affinity-purified antiserum against Vti1p and a monoclonal antibody against the HA epitope (16B12, Covance). Primary antibodies were detected using appropriate Cy2- or Cy3- conjugated secondary antibodies (Jackson ImmunoResearch).
