# Supplementary material for: Importance of the N-Terminal Domain of the Qb-SNARE Vti1p for Different Membrane Transport Steps in the Yeast Endosomal System
Source: PLoS One. 2013 Jun 12;8(6):e66304. doi: 10.1371/journal.pone.0066304 (PMC3680383; doi:10.1371/journal.pone.0066304)
Supplement: Figure S2 — GFP-Snc1p and DsRed-Sec7p localization at 24°C and 37°C in wild type and vti1-3 cells. (PDF) [file pone.0066304.s002.pdf]

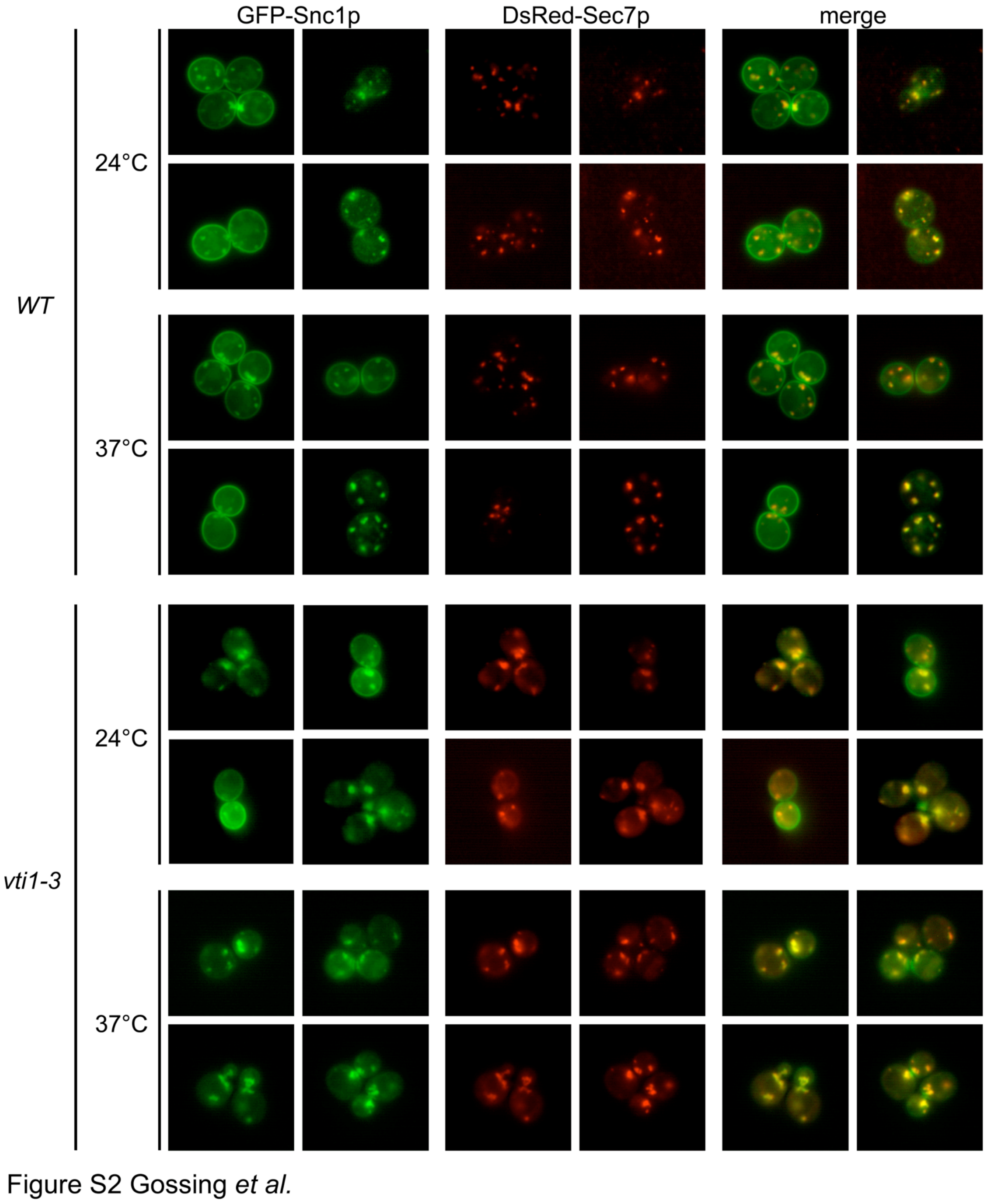

Figure S2. GFP-Snc1p and DsRed-Sec7p localization at 24°C and 37°C in wild type and *vti1-3* cells. In wild type and *vti1-3* mutant cells, the majority of GFP-Snc1p was localized to the plasma membrane at 24°C, while some GFP-Snc1p was detectable in intracellular structures which co-localized with DsRed-Sec7p. At 37°C, the majority of GFP-Snc1p was localized to the plasma membrane in wild type cells. In *vti1-3* cells, GFP-Snc1p accumulated in intracellular structures, which co-localized with DsRed-Sec7p at 37°C. This was accompanied by a complete loss of plasma membrane staining in most *vti1-3* cells. Strains used: SEY6210 pJJ8 pTPQ128 (WT), SCY14 pJJ8 pTPQ128 (*vti1-3*).
